# Endothelial and systemic upregulation of miR-34a-5p fine-tunes senescence in progeria

**DOI:** 10.18632/aging.203820

**Published:** 2022-01-12

**Authors:** Christina Manakanatas, Santhosh Kumar Ghadge, Azra Agic, Fatih Sarigol, Petra Fichtinger, Irmgard Fischer, Roland Foisner, Selma Osmanagic-Myers

**Affiliations:** 1 Max Perutz Labs, Center for Medical Biochemistry, Medical University of Vienna, Vienna Biocenter Campus (VBC), Vienna A-1030, Austria; 2 Institute of Medical Chemistry, Center for Pathobiochemistry and Genetics, Medical University of Vienna, Vienna A-1090, Austria

**Keywords:** Hutchinson-Gilford progeria syndrome, cardiovascular disease, endothelial senescence, senescence-associated micro RNAs

## Abstract

Endothelial defects significantly contribute to cardiovascular pathology in the premature aging disease Hutchinson-Gilford progeria syndrome (HGPS). Using an endothelium-specific progeria mouse model, we identify a novel, endothelium-specific microRNA (miR) signature linked to the p53-senescence pathway and a senescence-associated secretory phenotype (SASP). Progerin-expressing endothelial cells exert profound cell-non-autonomous effects initiating senescence in non-endothelial cell populations and causing immune cell infiltrates around blood vessels. Comparative miR expression analyses revealed unique upregulation of senescence-associated miR34a-5p in endothelial cells with strong accumulation at atheroprone aortic arch regions but also, in whole cardiac- and lung tissues as well as in the circulation of progeria mice. Mechanistically, miR34a-5p knockdown reduced not only p53 levels but also late-stage senescence regulator p16 with no effect on p21 levels, while p53 knockdown reduced miR34a-5p and partially rescued p21-mediated cell cycle inhibition with a moderate effect on SASP. These data demonstrate that miR34a-5p reinforces two separate senescence regulating branches in progerin-expressing endothelial cells, the p53- and p16-associated pathways, which synergistically maintain a senescence phenotype that contributes to cardiovascular pathology. Thus, the key function of circulatory miR34a-5p in endothelial dysfunction-linked cardiovascular pathology offers novel routes for diagnosis, prognosis and treatment for cardiovascular aging in HGPS and potentially geriatric patients.

## INTRODUCTION

Life expectancy has significantly risen in modern societies but this has been accompanied by an increase in age-related diseases such as cardiovascular diseases (CVDs), which are the leading cause of deaths globally (WHO report 2017) [1]. One of the key initiating events in age-related cardiovascular pathologies is endothelial dysfunction characterized by increased oxidative stress, reduced response to shear stress and consequently decline in atheroprotective endothelial nitric oxide synthase (eNOS). Increasing evidence also highlights the importance of deregulated microRNAs (miRs) in the development of endothelial dysfunction. Several studies demonstrated the involvement of circulating miRs in various age-related pathologies with a remarkable success of miR neutralizing antimiR agents in the treatment of age-related CVD [2, 3]. However, the tissue specific origins of age-related circulating miRs, particularly in regard to endothelial tissue *in vivo* and their mode and extent of action still remain widely unclear.

Age-linked tissue damage is often associated with cellular senescence occurring in almost all tissues [4, 5]. Cellular senescence occurs upon different cellular stressors such as DNA damage, mechanical damage or telomere erosion [6]. Cell cycle arrested, senescent cells can have deleterious effects on the surrounding cells through their metabolic activities, thereby playing a pivotal role in pathophysiological processes [7]. Senescent cells establish a “communication network” with their environment via secretion of pro-inflammatory and pro-fibrotic factors, growth factors and proteases, known as senescence-associated secretory phenotype (SASP) [8]. Moreover, pro-senescent effects can be mediated through paracrine transmission [9]. At the molecular level, the p53/p21 axis is a key pathway regulating senescence development [10]. p21 (*Cdkn1*) is a cyclin-dependent kinase inhibitor (CDKi) that acts in the early reversible stages of cell cycle arrest, whereas prolonged cell cycle arrest leads to the expression of another CDKi, p16 (*Cdkn2*) [10]. Persistent and high levels of p16 lead to irreversible permanent cell cycle arrest [11]. miRs are also emerging as key regulators of senescence and *bona fide* members of the SASP [12, 13]. miRs regulate gene expression at the post-transcriptional level through base pairing mainly to the 3’ untranslated regions of target messenger RNAs (mRNAs), leading to either mRNA decay or translational repression [14]. miRs that downregulate tumor suppressors act as oncogenes, those that downregulate oncogenes as tumor suppressors [15]. Consequently, deregulation of miRs is commonly found in cancer but also in aging and age-related diseases [15–18]. Several senescence-associated miRs, termed geromiRs, were proposed to be key components regulating senescence [19]. Among those, miR34a-5p was one of the first identified p53 targets and plays a key role within the p53-network [20]. From the clinical perspective, a large number of preclinical and clinical studies demonstrated elevated levels of miR34 in heart failure and therapeutic inhibition of the miR-34 family (miR-34a, -34b, -34c) attenuated the cardiac remodeling, left ventricular dysfunction and promoted human heart progenitor proliferation [21–23]. However, not much is known regarding the cell type specific impact of miR34 particularly at the organismal level which is an essential prerequisite for treating cardiovascular disease.

Given the potential importance of senescence-associated miRs and the involvement of a dysfunctional endothelium in age-related CVD pathology, we sought to explore whether an endothelial specific miR signature and alterations of circulating miRs may contribute to cardiovascular aging and pathology. To assess the potential effects of endothelium-specific senescence pathways and senescence-associated miRs on CVD pathology, we used an endothelium-specific mouse model of the premature aging disease Hutchinson Gilford progeria syndrome (HGPS), which develops many age-related CVD pathologies [24]. HGPS is caused by an autosomal dominant *de novo* (GGC>GGT) mutation in the *LMNA* gene that leads to activation of a cryptic splice site and generation of truncated and permanently farnesylated prelamin A, termed progerin [25, 26]. *LMNA* codes for A type lamins that together with lamin B form a mesh-like structure at the nuclear periphery providing mechanical stability to the nucleus and regulating chromatin organization and gene expression [27]. Progerin expression in cells leads to mechanical defects, lobulated nuclei and changes in heterochromatin, DNA damage and shortened telomeres, similar to aging-linked mechanisms [28]. Accordingly, HGPS patients develop many age-related features such as alopecia, skin scleroderma, lipodystrophy, bone abnormalities and prominent CVD within the first one to two decades of life [26]. Vascular smooth muscle cell (VSMC) loss in VSMC-specific progerin mice contributes to severe atherosclerosis, but recent studies in endothelium-specific progeria mice by us and others have also underpinned the importance of endothelial dysfunction in the development of age-related cardiovascular pathology in HGPS [24, 29, 30]. Dysfunctional endothelial cells show deregulation of the mechanoresponsive myocardin-related transcription factor A (MRTFA) and Sirt7 activity and exert pro-fibrotic and pro-inflammatory effects. In addition, several key endothelial-specific functions were also impaired in HGPS endothelial cells derived from induced pluripotent stem cells [31, 32]. However, the underlying mechanism and the potential involvement of senescence-associated miRs in endothelial cell dysfunction and its contribution to CVD are unknown.

Here, using the endothelium-specific progeria mouse model [24], we identify a novel endothelial-specific senescence-associated miR signature linked to a senescence-associated p53-signaling and systemic pro-senescent and pro-inflammatory SASP. Senescence-associated miR34a-5p that putatively affects 50% of downregulated mRNA targets in progerin-expressing endothelial cells was also elevated in plasma and significantly upregulated in lung and heart tissues and also in non-endothelial cell populations indicating systemic effects. Importantly, antimiR-mediated inhibition of miR34a-5p reduced the expression of p53 and late-stage senescence marker p16, highlighting the key role of miR34a-5p in sustaining senescence in endothelial cells and presumably through an unknown paracrine mechanism, also in neighboring non-endothelial populations. Thus, the current study identifies novel potential SA-miR mediated control mechanisms involved in senescence regulation and in vascular induced systemic aging effects in HGPS.

## RESULTS

### Progerin expression in endothelial cells activates p53-linked senescence and SASP

**Figure 1:**
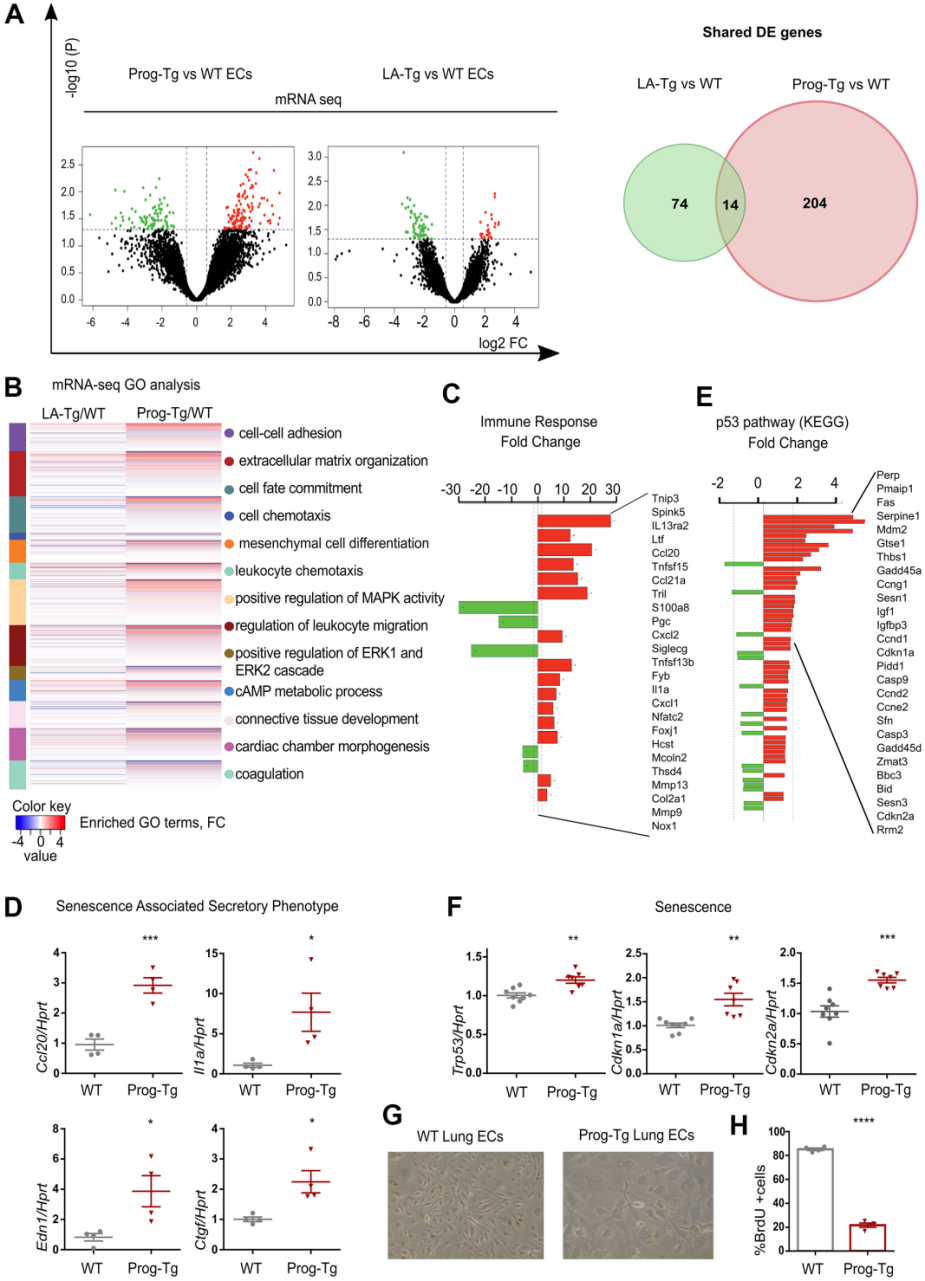
**Progerin expression in endothelial cells activates senescence and SASP.** (**A**) Volcano plots depicting differential expression (DE) analysis of genes in Prog-Tg/WT and LA-Tg/WT lung endothelial cells (ECs, left panel). Red, upregulated-, green, downregulated genes; X-axis, log_2_ values of fold change (FC>1,5 and <-1.5) and y-axis, -log_10_ values of p-value (p<0.05, n=3) (left panel). Analysis of shared DE genes between Prog-Tg/WT and LA-Tg/WT depicted by Venn diagrams (right panel). (**B**) Heatmap displaying enriched Gene Ontology (GO) terms of the whole ranked list of DE genes in Prog-Tg/WT and control group LA-Tg/WT using topGO and GOstats packages in R/Bioconductor. (**C**) Enriched GO term “Immune response” exhibiting significantly DE genes in Prog-Tg/WT lung ECs. (**D**) qPCR analysis of *Ccl20*, *Il1a*, *Edn1* and *Ctgf* in Prog-Tg/WT lung ECs using *Hprt* as reference gene. (**E**) KEGG pathway analysis of the “p53 pathway” enriched term showing DE genes in Prog-Tg/WT ECs. (**F**) qPCR analysis of *Trp53*, *Cdkn1a* and *Cdkn2a* in Prog-Tg/WT lung ECs using *Hprt* as reference gene. (**G**) Representative images from WT and Prog-Tg lung ECs at passage 2. (**H**) Bromodeoxyuridine incorporation assay (BrdU) performed for 40 h using Prog-Tg and WT lung ECs (n=4). For qPCR and BrdU assay, comparisons were performed between Prog-Tg and WT. For qPCRs n=4-8. Statistics were performed using unpaired two-tailed Students *t* test, *p<0.05, **p<0.01, ***p<0.001, ****p<0.0001.

In order to investigate molecular pathways underlying endothelial dysfunction in HGPS we used an endothelium-specific progeria mouse model (Prog-Tg) [24]. Prog-Tg mice were generated by crossing transgenic mice carrying a tet Operon (TetOp) driven human HGPS mutant lamin A minigene (TetOp-G608G) [33] with transgenic mice expressing a tetracycline-responsive trans-activator (tTA) under the control of the endothelium-specific VE-cadherin (*Cdh5*) promoter [34]. Prog-Tg mice were shown to express both human wildtype lamin A and mutant progerin from the transgene at similar levels [24, 33] (Supplementary Figure 1). As a control, we generated transgenic mice expressing the wildtype human lamin A (LA-Tg) using the same strategy (Supplementary Figure 1). We then performed transcriptome analysis of freshly prepared progerin-expressing endothelial cells (ECs) derived from these mice, and of control ECs derived from mice with transgenic expression of wildtype lamin A (LA-Tg) together with the corresponding littermate controls (WT) [24]. Differential expression analysis between Prog-Tg versus WT and LA-Tg versus WT ECs revealed 131 transcripts significantly up- and 87 downregulated in Prog-Tg versus WT, and evidently much less change in LA-Tg versus WT samples (25 up- and 63 downregulated) with only 14 shared differentially expressed (DE) genes (Figure 1A and Supplementary Table 1). The direct comparison of deregulated genes in Prog-Tg versus LA-Tg ECs showed 147 genes significantly up- and 44 downregulated (Supplementary Figure 2A and Supplementary Table 1) and a 3-way Venn diagram analysis revealed only a weak overlap between these sets of differentially expressed genes (Supplementary Figure 2B). This clearly indicates a unique effect of progerin expression on the EC transcriptome rather than lamin A overexpression per se. Heatmap clustering showed a high degree of correlation between the three biological replicates per genotype (Supplementary Figure 2C). Prog-Tg/WT transcriptome ranked-list enrichment analysis revealed a strong enrichment of the terms “cell-cell adhesion” (124 genes) and “extracellular matrix organization” (172 genes) consistent with previous reports [35]. Furthermore, the terms “leukocyte chemotaxis” (144 genes) and “regulation of leukocyte migration” (141 genes) indicate an inflammatory response, whereas “mesenchymal cell differentiation” (169 genes) and “connective tissue development” (199 genes) point to a pro-fibrotic response (Figure 1B and Supplementary Figure 3A, 3B). Lessened enrichment was observed for these pathways in the control LA-Tg/WT transcriptome (Figure 1B and Supplementary Figure 3A). Importantly, gene ontology (GO) analysis for significantly differentially expressed (DE) genes (FC>1,5 and < -1.5; p>0.05) in the Prog-Tg/WT transcriptome revealed among the top terms “immune response category” with 20 significantly deregulated genes including interleukin (*Il1a*) and many chemokines (Figure 1C). This term was not found in LA-Tg ECs. We confirmed the top upregulated inflammatory factors, CC chemokine ligand 20 (*Ccl20*) and interleukin 1a (*Il1a*), and key pro-fibrotic factors such as connective tissue growth factor *(Ctgf)* and endothelin *(Edn1)* in ECs derived from lung and heart tissues by quantitative real time PCR (qPCR). *Ccl20*, *Il1a*, *Edn1* and *Ctgf* were upregulated in Prog-Tg/WT lung ECs and *Ccl20* in Prog-Tg heart ECs (Figure 1D and Supplementary Figure 4A). However, no significant changes in the expression of these factors were detected in LA-Tg ECs (Supplementary Figure 4B, 4C). Since upregulation of pro-fibrotic and pro-inflammatory factors pointed towards the initiation of a senescence-associated secretory pathway (SASP) in Prog-Tg ECs, we checked for senescence-mediating signaling pathways by functional enrichment analysis against KEGG (Kyoto Encyclopedia of Genes and Genomes). This analysis identified significant enrichment of the p53-pathway comprising many direct p53 targets and senescence-associated genes (Figure 1E). qPCR analysis confirmed significant upregulation of *Trp53* (p53) and validated senescence-linked genes, cyclin-dependent kinase inhibitors *Cdkn1a* (p21^Cip1/Waf1^) and *Cdkn2a* (p16^Ink4a^) (Figure 1F). Accordingly, Prog-Tg primary lung ECs at passage 2 in culture showed an inflated cell phenotype, a distinctive characteristic of senescent cells (Figure 1G). Furthermore, the number of cycling cells was dramatically reduced in Prog-Tg EC vs WT EC cultures, as revealed by bromodeoxyuridine (BrdU) incorporation assay (Figure 1H). Thus, our data suggest that progerin expression in ECs activates p53-linked senescence and SASP with endothelial-specific immunomodulatory function and pro-fibrotic signaling. These data are in line with our previous study revealing perivascular and interstitial cardiac fibrosis in Prog-Tg mice [24].

### Cellular senescence and SASP *in vivo* in Prog-Tg mice

**Figure 2:**
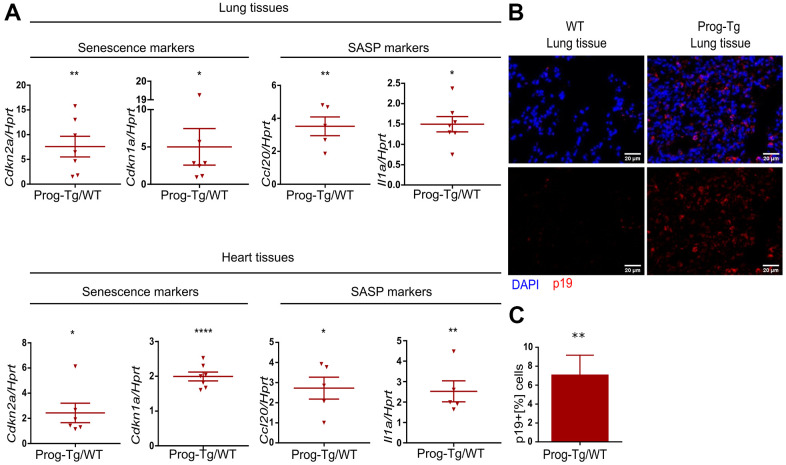
**Senescence and SASP are detected *in vivo* in Prog-Tg mice.** (**A**) qPCR analysis of depicted genes using RNA extracts from lung and heart tissues of Prog-Tg versus WT littermate mice (>25 weeks), n=5-7. (**B**) Representative immunofluorescence Z-stack projections from lung histological tissues sections of Prog-Tg and WT littermate mice at >25 weeks stained with anti-p19 antibodies and DAPI, Scale bar=20 μm. (**C**) Quantification of the percentage of p19-positive cells compared to DAPI-positive cells in lung tissues of Prog-Tg vs WT littermates on areas selected in a blinded manner (n=3). For qPCRs, a paired two-tailed Students *t* test was used and for statistic evaluation of histological staining a Mann-Whitney test. *p<0.05,**p<0.01, ****p<0.0001.

To examine senescence and SASP *in vivo* we first performed gene expression analysis in lung and heart tissues derived from Prog-Tg mice and corresponding WT littermates. Both senescence markers, *Cdkn1a* and *Cdkn2a*, were significantly upregulated in lung and heart tissues of Prog-Tg animals (Figure 2A). Concomitantly, inflammatory paracrine mediators *Ccl20* and *Il1a* were significantly increased in the lung and heart of Prog-Tg animals (Figure 2A).

Senescent cells upregulate p19^Arf^ (*Cdkn2d*), an upstream activator of p53 signaling and a valid senescence marker for histological analyses [36]. Immunofluorescence staining of lung tissues from ~25-week-old Prog-Tg animals showed a marked accumulation of p19^+^ cells compared to their WT littermates (Figure 2B). Quantitative analysis revealed an over six-fold increase in p19^+^ cells in Prog-Tg lung specimens, supporting upregulation of senescence *in vivo* (Figure 2C).

**Figure 3:**
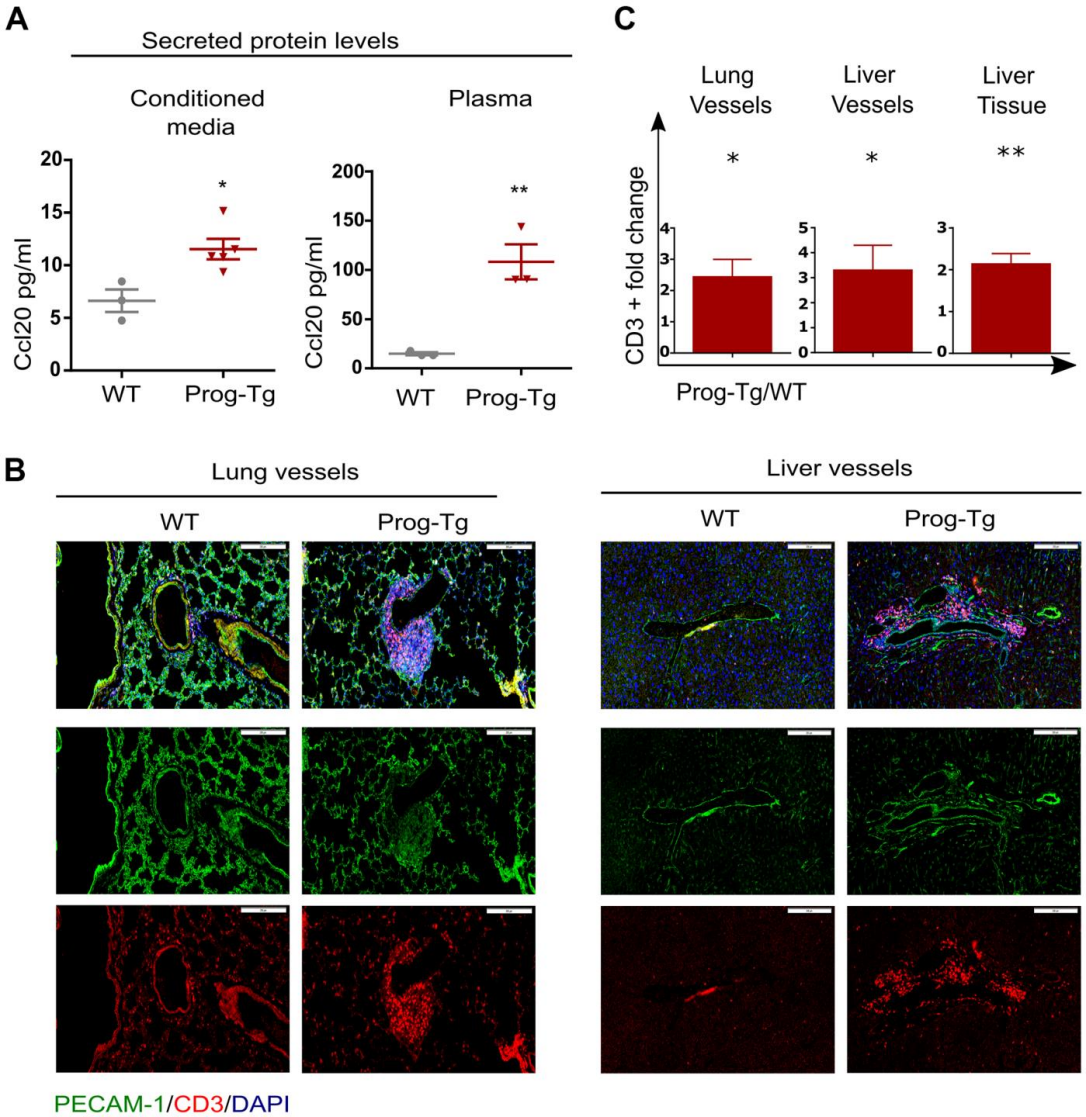
**Pro-inflammatory effects in Prog-Tg mice.** (**A**) ELISA was used to detect Ccl20 in cell culture supernatant samples of lung ECs and in plasma of ~30-week-old Prog-Tg and WT mice (n=3-4). (**B**) Representative immunofluorescence Z-stack projections of lung and liver sections from Prog-Tg and WT littermates (>25 weeks) stained with anti-PECAM and anti-CD3 antibodies and DAPI. (**C**) Quantification of CD3-positive cells in the vicinity of lung and liver vessels and in liver tissues counted in 10-15 independently selected areas in 4 pairs of Prog-Tg vs WT littermates. Scale bar=200 μm. For ELISA, an unpaired two-tailed Students *t* test was used. For statistic evaluation of histological stainings, a paired students two-tailed *t* test. *p<0.05, **p<0.01.

Senescent cells mediate pro-inflammatory effects through secretion of SASP factors such as Ccl20 and Il1a [9]. We therefore tested the levels of these proteins in conditioned media and plasma of Prog-Tg mice. Prog-Tg ECs secreted significantly higher levels of Ccl20 compared to WT ECs with even more dramatic effects in plasma samples showing a ~seven-fold elevation (Figure 3A). Importantly, the conditioned medium from control LA-Tg ECs and plasma samples from LA-Tg animals did not show an increase in Ccl20 levels (Supplementary Figure 4D). In contrast to the increase in Ccl20 levels in Prog-Tg plasma, we did not find detectable levels of Il1a, neither in conditioned media nor in plasma samples (data not shown). As Ccl20 is known to recruit immune cells, we were prompted to test immune cell infiltration in the lung of Prog-Tg mice and also liver tissue, which is a key site of immunological defense [9, 36]. Indeed, we found an accumulation of CD3+ immune rosettes surrounding PECAM-positive blood vessels in tissues of Prog-Tg mice (Figure 3B). Quantification of randomly chosen areas in the proximity of blood vessels in lung and liver tissue sections of Prog-Tg vs littermate controls revealed a ~two-fold increase in immune cell infiltration (Figure 3C). In liver tissue, the effect was even more pronounced with a ~three-fold increase in CD3+ infiltrates in the vicinity of blood vessels but also interstitially (Figure 3B, 3C). Altogether, these data suggest that progerin-expressing endothelial cells exert systemic effects on surrounding tissues through SASP.

### Progerin-expressing ECs exert paracrine senescence

**Figure 4:**
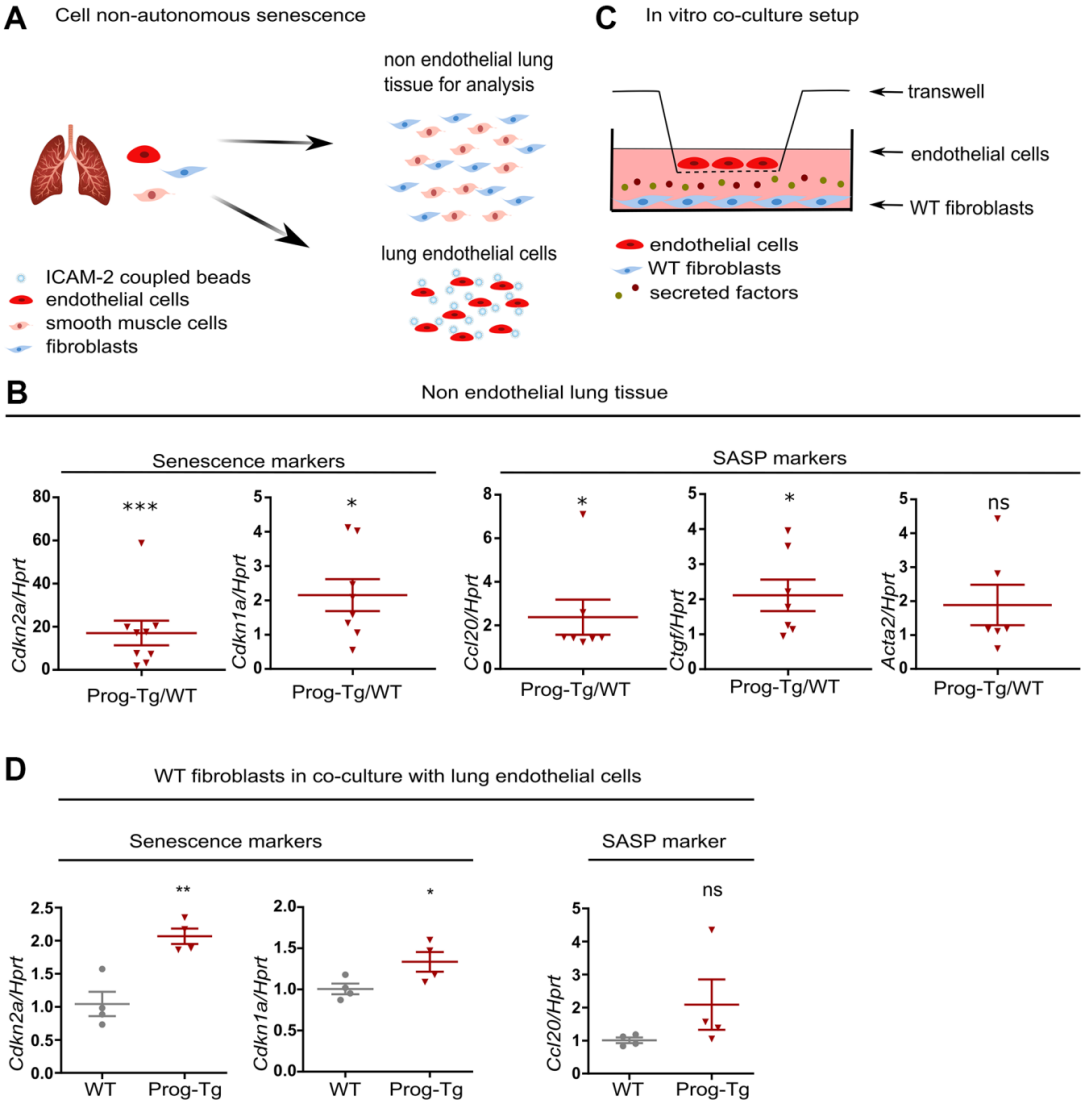
**Progerin expressing ECs exert paracrine senescence.** (**A**) Schematic representation depicting the separation of EC- from non-EC populations in lung tissue. (**B**) qPCR analysis of senescence and SASP marker genes in non-EC populations of WT vs Prog-Tg mice (>25 weeks). (**C**) Schematic representation of the *in vitro* co-culture setup. (**D**) qPCR analysis of senescence and SASP marker genes in WT fibroblasts co-cultured in the presence of WT or Prog-Tg ECs (n=4-9). Paired two-tailed Students *t* test was applied for *in vivo* experiments with littermate pairs and unpaired two-tailed Students *t* test for *in vitro* co-culture experiments. ns=non-significant, *p<0.05, **p<0.01, ***p<0.001.

Senescent hepatocytes were shown to induce inflammation and senescence in surrounding tissues in a paracrine fashion [9]. To examine the specific paracrine effect of progerin-expressing endothelial cells, we tested senescence and key SASP markers in non-endothelial cell populations obtained from lung tissues of Prog-Tg mice mostly depleted of endothelial cells (Figure 4A and Supplementary Figure 5A, 5B). Gene expression analysis revealed significant upregulation of key markers of senescence (*Cdkn2a* and *Cdkn1a)*, inflammation (*Ccl20)* and fibrosis (*Ctgf* and a trend for *Acta2)* in non-endothelial cell populations from Prog-Tg lung tissue (Figure 4B). These data strongly support the notion that progerin-expressing ECs induce senescence and elevate pro-inflammatory and pro-fibrotic effects in non-endothelial cell populations in a paracrine fashion in surrounding tissues.

Previously, we showed that Prog-Tg ECs exert pro-fibrotic paracrine effects on surrounding fibroblasts in an *in vitro* co-culture model system*.* The fibroblasts switched to *Acta2* expressing myofibroblasts in the co-culture with progerin-expressing ECs [24]. To directly assess if Prog-Tg ECs were also able to induce senescence and inflammation in a cell non-autonomous manner, we utilized the same set up with ECs seeded on transwell inserts placed on top of wells with cultured WT fibroblasts, allowing no physical contact between these cell types except through secretion of soluble factors (Figure 4C). In the presence of lung Prog-Tg ECs, fibroblasts express significantly higher levels of senescence markers *Cdkn1a* and *Cdkn2a* with a trend towards a pro-inflammatory phenotype as assessed by the expression of *Ccl20* (Figure 4D). Importantly, Prog-Tg ECs derived from heart tissues exert paracrine senescence and promote a myofibroblast switch in the co-culture system as assessed by an increase in *Cdkn2a* and *Acta2* levels compared to fibroblasts co-cultured with WT ECs (Supplementary Figure 5C). Altogether, these results demonstrate that progerin expression in endothelial tissue acts on different vascular beds such as lung and heart through paracrine senescence, fibrosis and inflammation, indicating systemic effects.

### Senescent progerin-expressing ECs display a specific senescence-associated miR signature

**Figure 5:**
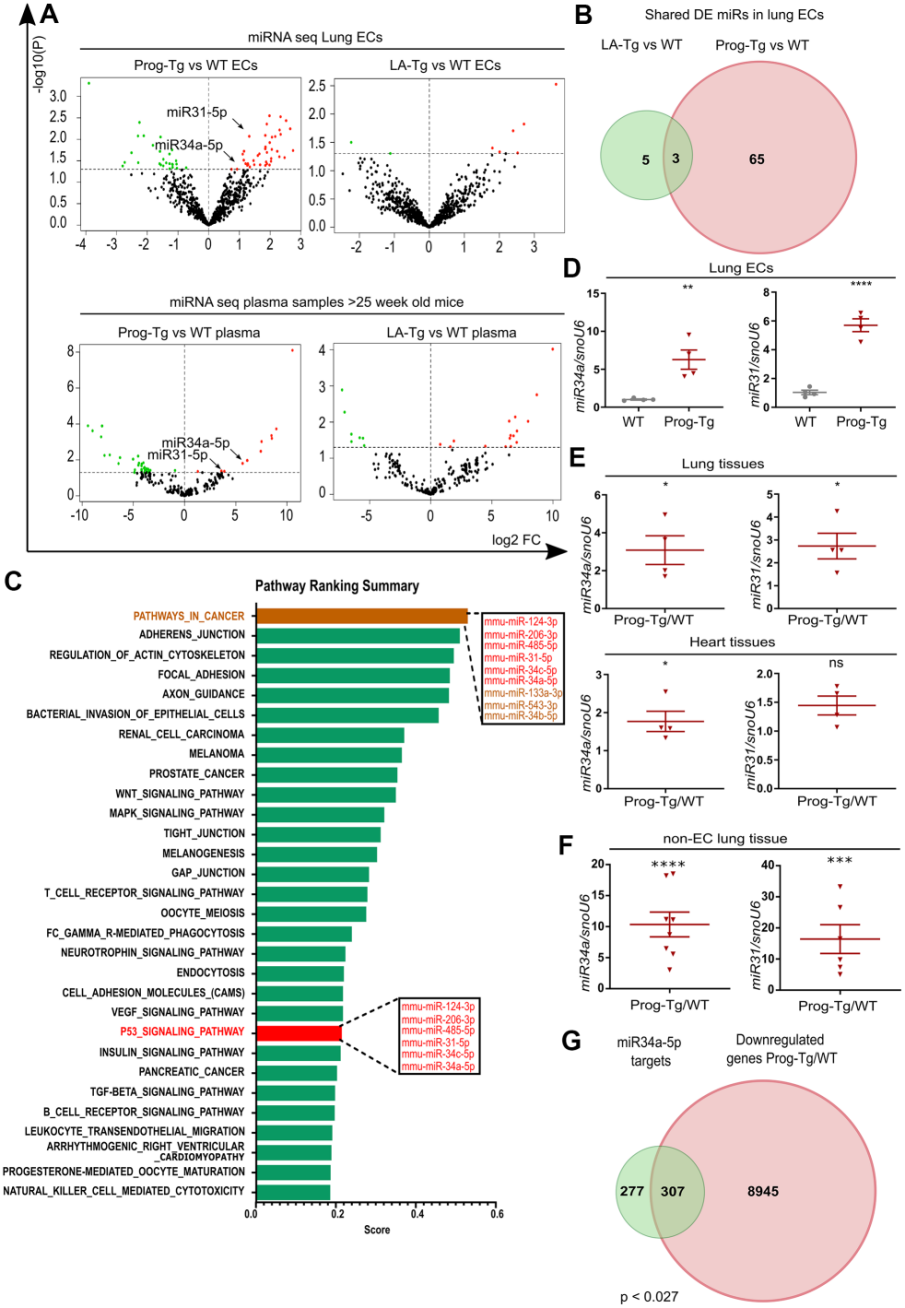
**Senescent progerin-expressing ECs display a specific senescence-associated miR signature.** (**A**) Volcano plots exhibiting differential expression (DE) analysis of miRs in Prog-Tg/WT and LA-Tg/WT lung ECs (upper panels) and in plasma samples of >25-week-old mice (lower panels) using threshold as depicted in Figure 1A (n=3). (**B**) Venn diagram showing overlap of DE miRs in Prog-Tg/WT and LA-Tg/WT lung ECs (topGO and GOstats packages in R/Bioconductor were used). (**C**) Bar graph representing the predicted top 30 pathways for the targetome of significantly DE miRs in Prog-Tg ECs with enriched term on the y-axis and ranking score on the x-axis. miRs linked to the “p53 signaling pathway” and “cancer pathway” are displayed in boxes (miRSystem version 20160513). (**D**) Expression levels analysed by qPCR of miR34a-5p (miR34) and miR31-5p (miR31) in cultured lung ECs normalized to small nuclear U6 RNA (snoU6). (**E**) Expression levels of miR34 and miR31 in the whole lung and heart tissues of Prog-Tg vs WT mice (>25 weeks) normalized to snoU6. (**F**) Expression levels of miR34a-5p and miR31-5p in non-EC populations from lung tissues of Prog-Tg vs WT mice normalized to snoU6. (**G**) Venn diagram displaying overlap between downregulated genes in Prog-Tg/WT lung ECs and miR34a-5p targets (Targetscan mouse) (Bioinformatics and Evolutionary Genomics). Hypergeometric test for the overlap showed a p-value of 0.027 and a representation factor of 1.1. For qPCRs n=4-8. The unpaired Students *t* test was used for *in vitro* experiments, paired Students *t* test for *in vivo* experiments using WT littermate controls. ns=non-significant, *p<0.05, **p<0.01, ***p<0.001, ****p<0.0001.

Growing evidence from recent reports highlights the importance of circulating microRNAs (miRs) in plasma of elderly individuals that pose a high risk for cardiovascular incidents, however, with only a few reports on their cell type-specific origin and functions studied in *in vivo* animal models [2, 3, 12]. Thus, we explored the endothelial-specific miR signature with potential senescence regulatory function and ability to exert systemic effects using miR expression profiling in isolated endothelial cell extracts and plasma obtained from aged Prog-Tg and corresponding control animals. 68 and 51 differentially expressed (DE) miRs were found in ECs and plasma samples from Prog-Tg mice, respectively. Only 3 commonly shared deregulated miRs were found between Prog-Tg/WT and LA-Tg/WT lung ECs, indicating a progerin-specific effect on miR expression (Figure 5A, 5B and Supplementary Table 2). A comparative targetome KEGG pathway enrichment analysis of 41 significantly upregulated miRs conducted by miRSystem revealed the enrichment for the terms “cancer pathway” and “p53 signaling pathway” with deregulated miRs 124-3p, 206-3p, 485-5p, 31-5p, 34c-5p and 34a-5p (Figure 5C). This is conceivable since p53 signaling plays a key reciprocal role in cancer and senescence regulation [20]. The linkage of these miRs to the p53 pathway, together with previous findings on their upregulation during senescence [19, 37, 38] suggested that they fulfil features of senescence-associated (SA) miRs [12] and may thus represent an endothelial specific SA-miR signature.

We next overlapped the deregulated miRs in ECs with those in plasma of Prog-Tg mice. Only two miRs, miR34a-5p and miR31-5p were upregulated in ECs as well as in plasma of Prog-Tg mice but not in LA-Tg mice, and both were enriched within the p53-signaling cluster (Figure 5A, 5C, 5D). In further support of their potential involvement in systemic effects, miR34a-5p and miR31-5p were found significantly upregulated in whole lung tissue (Figure 5E) as well as in the non-EC lung populations depleted of ECs and in lung derived ECs (Figure 5F and Supplementary Figure 5D). Importantly, miR34a-5p but not miR31-5p was also found significantly upregulated in heart tissue (Figure 5E), implying the key importance of this miR in cardiac tissue and CVD pathology of Prog-Tg ECs. Finally, a strong trend towards increased expression of miR34a-5p and miR31-5p was observed in fibroblasts co-cultured in the presence of lung Prog-Tg ECs presumably due to either paracrine effects or direct secretion and uptake of these miRs (Supplementary Figure 5E). Overlapping analysis of putative miR34a-5p target mRNAs (using miR target prediction tool Targetscan), with all downregulated genes in Prog-Tg ECs (ranked list) revealed 307 commonly downregulated mRNAs, representing 53% of all known miR34-5p targets (Figure 5G). This suggested, in addition to the systemic also a cumulative effect of this miR on several targets in the Prog-Tg transcriptome.

### miR34a-5p fine-tunes senescence in progerin-expressing ECs

**Figure 6:**
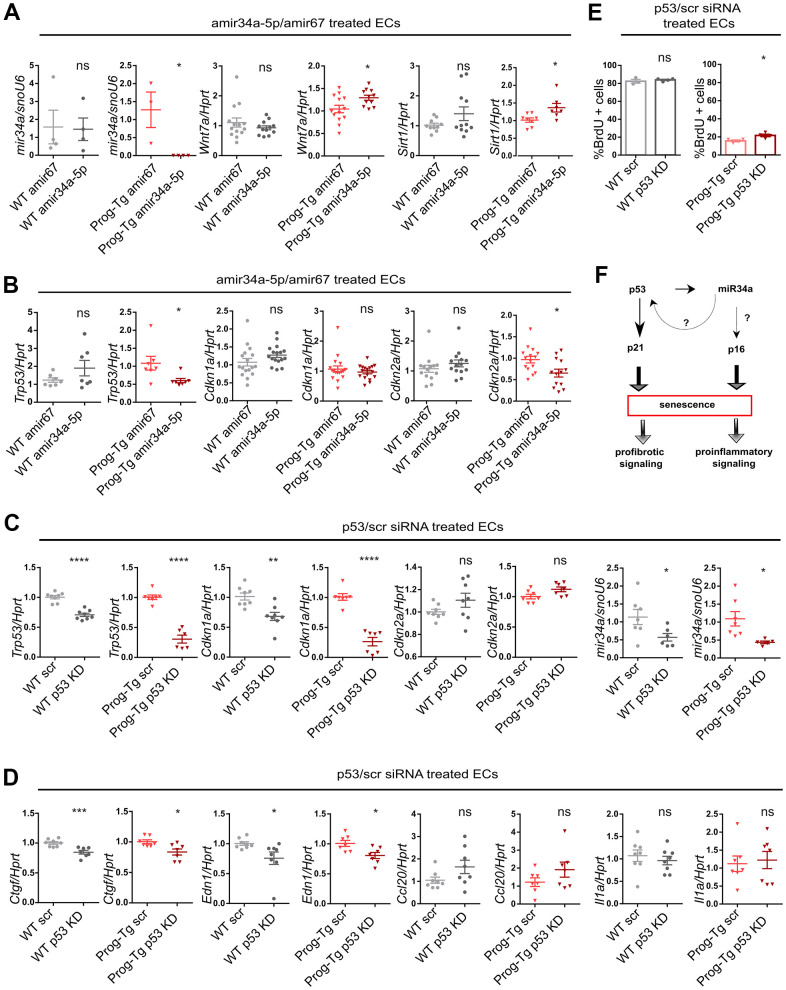
**miR-34a-5p fine-tunes senescence in progerin-expressing ECs.** WT and Prog-Tg lung ECs were transfected with antimiR34a-5p (amir34a-5p) or control antimir67 (amir67) and tested for (**A**) expression levels of miR34a-5p, and mir34 targets *Wnt7a* and *Sirt1* and (**B**) *Trp53*, *Cdkn1a* and *Cdkn2a*. WT and Prog-Tg lung ECs were transfected with p53 neutralizing siRNAs or scrambled siRNA (scr) and tested for (**C**) gene expression levels of senescence markers *Trp53*, *Cdkn1a, Cdkn2a* and miR34a-5p and (**D**) pro-fibrotic markers *Ctgf*, *Edn1* and pro-inflammatory markers (*Ccl20*, *Il1a*). (**E**) BrdU assay performed over 40 h. (**F**) Schematic representation of miR34a-5p acting on p53- and p16-branch regulating senescence pathways in Prog-Tg ECs. For qPCRs n≥3. Unpaired two-tailed Students *t* test, ns=non-significant *p<0.05, **p<0.01, ***p<0.001, ****p<0.0001.

Given the above observations implying a systemic relevance of miR34-5p and its potential involvement in CVD pathology in Prog-Tg mice, we next assessed whether the increased levels of miR34-5p affect the senescence phenotype of Prog-Tg ECs. For this, freshly isolated endothelial cell cultures were transfected either with antimiR neutralizing miR34a-5p or a control non-targeting antimiR. Specific antimiR treatment of Prog-Tg cells reduced the levels of miR34a-5p, as expected, and de-repressed the expression of its known direct targets, *Wnt7* and *Sirt1,* which were found significantly downregulated in Prog-Tg versus WT ECs, confirming the success of the antimiR treatment (Figure 6A and Supplementary Figure 6A, 6B). In WT cells, antimiR treatment did not affect the levels of miR34a-5p nor those of its target genes, probably due to the very low levels of miR34a-5p expressed in WT cells (Figure 6A and Supplementary Figure 6C). Importantly, miR34 inhibition downregulated p53 (*Trp5*3) but also significantly repressed the late-stage senescence regulator p16 (*Cdkn2*) (Figure 6B) in Prog-Tg, but not in WT cells, indicating its key role in maintaining the senescence phenotype in progerin-expressing ECs. In contrast, no effect was observed on early-stage senescence marker p21 *(Cdkn1)* and other SASP factors (Supplementary Figure 6D), suggesting the need for additional factors or more prolonged treatment for a fully resolved senescence phenotype. Since miR34a-5p was reported to act within the p53-signaling network [20], we next used p53 neutralizing siRNA to knockdown p53 to discern the p53 from the miR34a-5p-mediated effect on senescence and SASP. Compared to the scrambled siRNAs control, the p53-specific siRNA downregulated p53 (*Trp53*) levels by 70% in Prog-Tg ECs (Figure 6C). Similar to the results above, the p53-specific siRNA exerted only subtle effects in WT cells, presumably due to very low levels of *Trp53* in these cells (Figure 6C). In contrast to antimiR34a-5p treatment, p53 knockdown had no effect on p16 (*Cdkn2a*) but profoundly reduced the levels of its direct downstream targets, senescence marker p21 (*Cdkn1a*) and miR34a-5p, as expected and partially ameliorated the endothelial-specific SASP as evident by reduced levels of pro-fibrotic factors *Ctgf* and *Edn1* (Figure 6C, 6D), suggesting complementary effects of p53 and miR34a-5p in senescence regulation. Again, WT cells showed a similar but mostly reduced effect upon treatment (Figure 6C, 6D). The significant reduction in p53 and p21 levels in Prog-Tg cells correlated with a subtle but significant increase in proliferation as measured by BrdU incorporation assay (Figure 6E). Overall, these data suggest that miR34a-5p regulates senescence in endothelial cells by acting on two separate senescence signaling branches, a well-established p53- and, in addition a p16 pathway with presumably systemic effects on surrounding non-endothelial cell populations (Figure 6F).

### Senescence and accumulation of miR-34a-5p at atheroprone aortic arch regions of Prog-Tg mice

**Figure 7:**
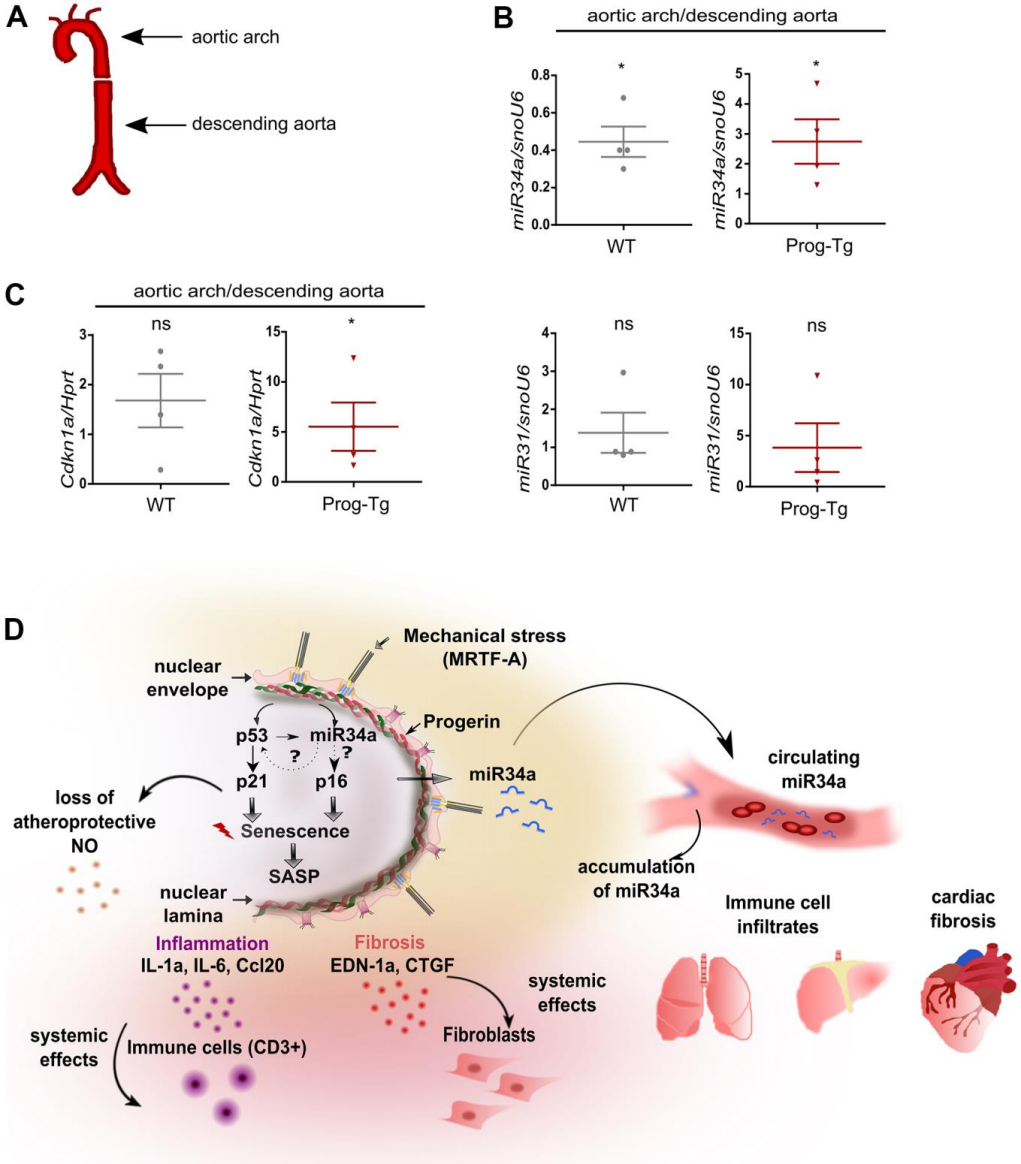
**Analysis of circulatory SA-miRs at atheroprone aortic arch regions.** (**A**) Schematic representation of aortic segment including aortic arch region and that of descending aorta. (**B**) Gene expression analysis of miR34a-5p (miR34a) and miR31-5p (miR31) and of (**C**) p21 (*Cdkn1a*) in WT and Prog-Tg aortic arch in comparison to descending aorta. n=4. Paired two-tailed Students *t* test, ns=non-significant *p<0.05. (**D**) Hypothetical model of intrinsic and extrinsic miR34-mediated senescence regulation in progerin-expressing endothelial cells. Mechanical stress particularly at vessel bifurcation leads to p53-linked senescence and miR34 upregulation. miR34 sustains senescence through positive feed-back mechanism acting on p53 but also separately by maintaining high levels of late senescence marker p16. Synergistic miR34-p53 action leads to elevation of SASP signaling and thus secretion of pro-inflammatory (Ccl20, IL-1a) and pro-fibrotic (CTGF, EDN-1a) factors with systemic effects on surrounding tissues leading to immune cell infiltrates and fibrosis in lung and liver and cardiac tissue. Systemic fibrosis and inflammation in tissues is further potentiated by increased release of miR34 in circulation.

Senescent cells have been shown to accumulate at atheroprone regions of impaired shear stress in aortic arch, thereby promoting the development of cardiovascular pathologies [39]. Given our previous findings of impaired shear stress in ECs- and aorta of Prog-Tg mice [24], we next examined if changes in senescence-associated miRs can be observed in different flow regions of aorta, which would have relevance to aging-linked cardiovascular pathology in these mice. For this, we dissected aorta from aged Prog-Tg and littermate mice and separated the upper region containing the aortic arch with disturbed blood flow patterns from that of descending aorta with laminar flow as previously reported [40] (Figure 7A). The distribution of senescence-associated miR34a-5p and miR31-5p between these aortic regions was then assessed by gene expression analysis of the corresponding tissue extracts. We found significant upregulation of miR34a-5p and a trend towards higher levels of miR31-5p in the aortic arch regions in comparison to those of descending aortas in Prog-Tg mice, but not in littermate controls, that correlated well with upregulation of p21 senescence marker (*Cdkn1*) (Figure 7B, 7C). These data indicate that mechanical stress, which is particularly high at regions of vessel curvatures such as in the aortic arch, leads to increased mechanical damage, senescence and increased expression of endothelial senescence associated miRs. Thus, particularly the circulating SA-miR34a-5p, which may putatively mediate systemic effects in non-endothelial surrounding cell populations, could be a potential target for clinical studies in the treatment of endothelial-rooted cardiovascular defects (Figure 7D).

## DISCUSSION

microRNAs have recently gained immense attention in the treatment of various age-related cardiovascular pathologies [3]. However, precise miR-mediated treatments require the identification of specific cell type-mediated defects and elucidation of the accompanying miR signature and its functions. In this study, we identify an endothelial-specific miR-signature involved in p53-linked senescence with accompanying pro-inflammatory and pro-fibrotic systemic effects contributing to cardiovascular aging pathology in HGPS.

Cellular senescence has emerged as a primary driver of age-related pathologies, as senescent cells were shown to accumulate at regions of cellular insults such as atherosclerotic plaques particularly at vessel bifurcations. In line with this, the elimination of senescent cells reduced atherogenesis and cardiac aging [39, 41]. Similarly, a growing number of studies delineate the importance of endothelial senescence in developing age-related cardiovascular pathologies [1]. However, there is limited evidence on the specific-miRs and their role in endothelial senescence studied *in vivo* and to what extent these provoke tissue deterioration. To address these questions, we used a HGPS mouse model with endothelium-specific expression of progerin. Our previous findings showed that these mice recapitulate many aspects of cardiovascular pathology of HGPS patients such as perivascular and interstitial fibrosis and diastolic dysfunction [24]. Similar cardiovascular pathologies were observed later also in another endothelium-specific progerin-expressing mouse model generated via a different strategy, which, in addition, exhibited significantly compromised acetylcholine-regulated vasodilation of thoracic aorta [30]. Both reports strengthen the pivotal role of endothelial dysfunction in the cardiovascular pathology of HGPS.

In order to identify the endothelial specific miR profile with potential novel mechanistic insights into endothelial dysfunction in progeria, we applied mRNA and miR transcriptome analysis of primary progerin-expressing ECs and plasma of aged Prog-Tg animals coupled with qPCR and histological analysis. We found deregulation of a plethora of endothelial specific miRs with peculiar upregulation of senescence-associated miRs, miR206-3p, miR124-3p, miR485-5p, miR31-5p and miR34a-5p and miR34c-5p together with upregulation of a p53-linked senescence pathway and initiation of a SASP phenotype. Among these p53-linked miRs, circulatory miR34a-5p has the ability to exert systemic effects. Using antimiR34a-5p and p53 knockdown approaches, we demonstrate that a complementing and synergistic action of p53 and miR34a-5p fine-tunes senescence in progerin-expressing ECs. The involvement of the p53/p21 in HGPS has also been proposed in a previous study [42], showing that the p21 (*CDKN1A*) gene is upregulated by epigenetic pathways upon progerin-induced oxidative stress in HGPS patient fibroblasts. Thus, the induction of senescence signaling in HGPS may be regulated through various pathways and at different levels synergistically.

Previous studies on senescent hepatocytes reported beneficial but also deleterious systemic effects on the surrounding tissue through a cell non-autonomous mechanism causing “bystander senescence” in normal non-senescent surrounding cells [9]. However, similar studies with senescent endothelial cell populations studied in a specific, isolated fashion *in vivo,* and the extent and the mode of action of these cell non-autonomous effects are still missing. Here we show marked upregulation of senescence not only in isolated EC cultures but also in whole heart and lung tissues and in endothelial cell-depleted lung populations of Prog-Tg animals indicating systemic effects. We can demonstrate the direct adverse effects of these senescent progerin-expressing ECs on their non-endothelial neighbors transmitting senescence and fibrosis in a paracrine fashion using co-culture cell model systems. Importantly, similar pro-senescent and pro-fibrotic effects detected in co-culture using heart-derived progerin-expressing ECs highlight the relevance to cardiovascular pathology but also indicate that this is a general phenomenon applicable to endothelial cells originating from different vascular beds.

Pro-senescent effects of senescent cells may have beneficial tumor suppressive effects, but during aging they have deleterious effects inducing chronic damage [43]. The mediators of either paracrine senescence or apoptosis were shown to be pro-inflammatory factors such as interleukin Il1a that triggers secretion of a plethora of immune factors such as different CC chemokine ligands or tumor necrosis factor 1α [9, 44]. The expression of pro-inflammatory mediators in HGPS was shown to be elicited by NF-κB and STAT1-mediated inflammatory responses rooted in DNA damage [45, 46]. Accordingly, anti-inflammatory treatments have been very successful in the treatment of HGPS [45–47]. In line with these findings, we found the “immune response term” as one of the uppermost enriched gene ontology (GO) terms among the significantly differentially expressed genes in progerin-expressing ECs. The immune response term comprises 20 significantly deregulated pro-inflammatory mediators and represents a specific endothelium-specific inflammatory signature. Here we could validate the systemic inflammatory effects of endothelial cells *in vivo* by showing upregulation of top-most immune response candidates, *CCl20* and *Il1a* in the lung and heart tissues and corresponding ECs and also circulating in plasma, in line with dramatic immune cell infiltrates in the vicinity of blood vessels in lung and liver tissues of Prog-Tg mice. The latter observation is in accordance with the primary function of pro-inflammatory mediators in recruiting immune cells with an aim to initiate immune-mediated clearance of damaged cells [43]. Senescent cells, however, have developed mechanisms to evade immune-mediated clearance resulting in the persistence of immune cell infiltrates in the proximity of senescent cells [48]. Altogether our findings indicate that persistent senescence of ECs and the induced inflammatory response was sufficient to cause immune-cell tissue infiltration and accumulation in the vicinity of blood vessels.

In recent years miRs have gained attention as potent regulators of gene expression in accelerated aging disease and during physiological aging [16]. The miR interactome is very complex since each miR has the ability to affect hundreds of mRNA targets and thus have multifunctional effects [16]. Vice versa, a specific mRNA can be regulated by several miRs in a context-dependent manner. Senescence associated (SA) miRs, also called geromiRs, were found upregulated upon onset of cellular senescence, affecting predominantly genes involved directly or indirectly in cell cycle regulation [12, 19, 37]. SA-miRs seem to act predominantly through their effect on the p53/p21 or p16/Rb signaling axis [37]. Within the p53/p21 axis, activated p53 has the ability to either directly act on miR promoters or through modulation of the miR processing Drosha complex, which both lead to increased expression of several miRs with tumor suppressive function [49, 50]. Hence, p53 activation is typically associated with upregulation of SA-miRs. Here we found upregulation of SA-miRs, miR-206, miR-124, miR34a-5p, miR34c-5p and miR31-5p, linked to p53 signaling but did not observe changes in miR-29 and miR-365, which were previously found deregulated in fibroblasts of Zmpste24-/- progeroid mice [17, 18]. Therefore, we conclude that these deregulated miRs resemble a unique endothelium-specific senescence-associated miR signature that will be a valuable tool for future clinical studies.

Among the upregulated SA-miRs, we focused on miR34a-5p, which was uniquely elevated in circulation as well as in lung and heart tissues of Prog-Tg mice. miR34a-5p was previously shown to be induced in human heart failure and animal models implicated in cardiovascular disease progression, and targeting the miR34a-5p has harnessed the therapeutic benefits in cardiac repair [21–23]. However, endothelial cell type specific miR34 effects in this context have not been investigated so far. Thus, these previous findings together with our observation of strong miR34a-5p accumulation at atheroprone regions of the aortic arch in Prog-Tg mice suggests that miR34a-5p is particularly relevant for cardiovascular pathology in HGPS and supposedly geriatric patients, with strong potential to be a novel predictive and therapeutic biomarker for CVD rooted in endothelial dysfunction. In addition to elevation in plasma and tissues, miR34a-5p levels were also dramatically upregulated in EC-depleted non-endothelial cell populations indicating potential systemic effects. miR34a-5p is a direct target of p53 and exerts tumor suppressive function via its repressive role on a plethora of targets involved in cell-cycle regulation. One study showed that it affects one quarter of mRNA targets within the p53 network [20, 49, 50]. One of the common miR34a-5p targets is NAD-dependent deacetylase silent information regulator 1 (*Sirt1*) that inhibits p53 through deacetylation and negatively affects cellular senescence [20]. We observed a modest decrease of *Sirt1* in Prog-Tg ECs, and *Sirt1* was significantly de-repressed upon anti-miR34 treatment. Another strongly downregulated miR34a-5p target in Prog-Tg/WT ECs, Wingless 7a (*Wnt7a*), was also de-repressed upon anti-miR34 treatment. The Wnt-pathway was previously found deregulated in HGPS and in physiological aging [51–53] and is one of the major pathways predicted to be affected by SA-miRs [16]. Here we show that de-repression of *Wnt7* and *Sirt1* correlates with a reduction of the late-senescence marker p16. Low to moderate grade damage usually leads to transient elevation of p21 to induce cell cycle arrest and, if the damage is repaired, is reverted to normal levels [10]. Consistent with this, we show that lowering p21 levels through siRNA mediated p53 knockdown could restore cell growth with no effect on p16 state. p16 is a late-stage senescence marker elevated upon increased persistent cellular stress, eventually causing permanent cell cycle arrest [10]. We hypothesize that increased miR34a-5p levels may contribute to persistent cellular stress through cumulative cell-cycle repression of *Sirt1* and *Wnt7* but also through the effects on many potential other targets, leading to permanent cell cycle arrest. The latter assumption is based on our Targetscan based prediction approach showing 50% of miR34a-5p targetome downregulated in Prog-Tg transcriptome. The novel finding that anti-miR34a-5p treatment is able to significantly lower p16-linked senescence in addition to p53-levels with minimal, non-significant reduction of p21 levels suggests that miR34a-5p mainly affects already cell-cycle arrested cells on their way to irreversible p16-mediated cell cycle arrest.

As for the underlying cause for the upregulation of miR34a-5p and the initiation of miR-linked p53 senescence we hypothesize that impaired shear stress and mechanical damage may be one of the triggering mechanisms (Figure 7B), because our previous findings revealed an impairment of the shear stress response and myocardin-related transcription factor A (MRTF-A) mechanosignaling pathway in progerin-expressing endothelial cells [24]. We addressed this potential model by testing miR34a-5p levels in curved regions of the aorta prone to disturbed blood flow and higher shear stress compared to the descending aorta exposed to laminar blood flow. In line with our hypothesis, we found accumulation of miR34a-5p and increased p21 levels particularly at the aortic arch region, in accordance with previous reports that disturbed flow, such as at aortic curvature, leads to activation of senescence signaling in endothelial cells [54, 55]. Thus, although our gene expression studies were performed in unstimulated cells without additional exposure to shear stress *in vitro*, the observed changes in gene expression in primary endothelial cells may still reflect the *in vivo* conditions of increased mechanical stress caused by progerin accumulation.

We found miR34a-5p levels also dramatically upregulated in plasma of Prog-Tg mice, indicating potential systemic effects. Therefore, progerin-expressing endothelial cells have the potential to affect a variety of tissues through secretion of miRs and other signaling molecules, such as adipose tissue [56]. Indeed, in Prog-Tg mice we observe loss of adipose tissue (our unpublished data) but mechanistic details remain to be explored in future studies.

Based on the novel findings in this study we propose the following hypothetical disease mechanism to explain EC-mediated cardiovascular pathology in HGPS (Figure 7D): Progerin induces persistent mechanical stress leading to the activation of the p53 pathway, and its target miRs, such as miR34-5p, rise particularly at mechanosensitive atheroprone vessel bifurcations. Presumably through EC-mediated secretion, miR34a-5p reaches the circulation causing systemic cell cycle repressive effects on the neighboring non-endothelial cell populations. This pro-senescent miR34a-5p-mediated effect in turn exacerbates SASP-mediated inflammation in target tissue such as lung and liver, and fibrosis of cardiac tissue. Mechanistically, on both the extrinsic- and the intrinsic endothelial level, miR34a-5p has the ability to repress several positive cell cycle regulators causing a gradual increase in p16 levels. If the damage and miR34a-5p/p16 levels persist, cells enter a p16-mediated irreversible permanent cell cycle arrest. As a consequence, senescent cells switch to a pro-inflammatory and pro-fibrotic secretome resulting in immune cell attraction and fibroblast to myofibroblasts switch, respectively. Specifically, in endothelial cells SASP counteracts the effects of healthy atheroprotective nitric-oxide containing secretome consistent with previous findings of low nitric oxide levels in progerin-expressing endothelial cells [24, 31, 32] (Figure 7D). Collectively, our data demonstrate that presumably through its key strategic position, endothelial senescence exerts systemic tissue deterioration, and part of this systemic cell-cycle repressive effect involves SA-miRs, particularly miR34a-5p. Fine-tuned senescence-associated antimiR therapies targeting specifically ECs could be a beneficial strategy for the treatment of endothelial senescence in premature and pathophysiological aging and as a complementary intervention strategy during cancer therapy-induced senescence.

## MATERIALS AND METHODS

### Animals

Bi-transgenic LA-Tg and Prog-Tg mice were generated *de novo* by crossing tet operon-driven transgenic lamin A minigene wildtype (*tetop-LA^Wt^*) or HGPS mutant (1824C>T; G608G; *tetop-LA^G608G^*) mice [33] (see Supplementary Figure 1, C57BL/6J background) with transgenic mice expressing a tetracycline-responsive transcriptional activator under the control of the EC-specific VE-cadherin promoter (*Cdh5-tTA* mice, Jackson Laboratories, MGI: 4437711, *FVB* background). The VE-cadherin (*Cdh5*) promoter includes the full promoter region (-2226, +24) as described [34], and is active exclusively in the endothelial tissue from embryonic day 13.5 (E 13.5) on [57]. Mice were maintained at C57BL/6J background (N4 generation; ~94%) as previously described [24]. Animal studies were approved by the Regional Ethics Committee for Laboratory Animal Experiments at the Medical University of Vienna and the Austrian Ministry of Science Research and Economy (BMWFW-66.009/0321-WF/V/3b/2016 and BMWFW-66.009/0156-WF/V/3b/2017) according to Austrian Law BGBI. I Nr.114/2012 (TVG2012) and in accordance with the Guide for the Care and Use of Laboratory Animals published by the US National Institutes of Health (NIH Publication No. 85-23, revised 1996).

### Primary endothelial cell isolation and culture

Primary endothelial cells were isolated from lungs and heart tissues of ~10-day old Prog-Tg and LA-Tg mice and corresponding WT littermate animals using ICAM-2 magnetic bead separation as described previously [24]. Briefly, lung tissue was isolated from mice, minced and digested in 200 U/ml collagenase type I (Gibco 17100-017) for 45 minutes at 37° C on an end over end rotor. For heart tissue, collagenase type II (Gibco 17101-015) was used. Thereafter, collagenase digested tissues were passed through an 18-gauge syringe needle 10-15 times and filtered through a 70μm Nylon cell strainer (Corning, REF: 431751). Cells were pelleted at 200 x g for 5 min and plated on 2% gelatin / 1 μg/ml fibronectin-coated plates. Cells were cultured in DMEM supplemented with 20% FCS, EC growth supplement (CellBiologics Catalog No 1166), 25 mM HEPES, 50 U/ml penicillin, 50 μg/ml streptomycin, 2 mM L-glutamine, 1 mM nonessential amino acids, 1 mM sodium pyruvate, and 139 μg/ml heparin (complete culture medium). Typically, after 48 hours, cells were incubated for 5 min at 37° C in a trypsin-EDTA solution (Sigma, T4049), resuspended in 1ml of cold medium and mixed with 10μl magnetic Dynabeads (Dynabeads Sheep Anti-Rat IgG Catalog No. 11035) coupled to ICAM-2 antibody (CD102 Rat anti-Mouse, Fisher Scientific REF: 553326). Cells were incubated for 45 min at 4° C and thereafter endothelial cells bound to the ICAM-2 coupled Dynabeads were separated from the rest of cells using a magnetic stand. Separated endothelial cells were cultured on a gelatin/fibronectin coated plate. Primary ECs were used for experiments at passage p2 or p3 after isolation.

### Co-cultures

Co-cultures experiments were performed as described previously [24]. ECs at passage 2 were seeded on cell culture transwell filter inserts (Costar Corning Incorporated, 3460; pore size 0.4 μm) at a density of 3.6 × 10^4^ cells/cm^2^. WT fibroblasts, isolated from lung tissue of neonatal C57BL/6J mice [24], were seeded on the bottom of 12-well cell culture plates at a density of 2.4 × 10^4^ cells/cm^2^. Cells were cultured separate for 24 hours and thereafter co-cultured for 4 days prior to RNA isolation. Extrinsic effects of ECs on co-cultured fibroblasts were assessed by expression analysis of the known pro-fibrotic factors *Acta2*, senescence markers *Cdkn1a* and *Cdkn2a* and pro-inflammatory marker *Ccl20*.

### AntimiR treatment

Primary lung ECs were transfected with an antimiR for miR34a-5p (miRIDIAN microRNA Hairpin Inhibitor assay from Dharmacon IH-310529-08-0005). Briefly, lung ECs of passage 2 were seeded on a 48-well cell culture plate at a density of 5×10^4^ cells/cm^2^ and cultured for 24 h in antibiotic free medium. Thereafter, cells were co-transfected with 100 nM antimiR34a-5p and 50 nM fluorescently labelled control antimiR (miRIDIAN microRNA Hairpin Inhibitor Transfection Control with Dy547 CP-004500-01-05) for 48 h using a lipofectamine-based transfection reagent (LipoFectMax transfection reagent ABP Biosciences FP310). As a negative control, the *C. elegans* Cel-miR-67 that has minimal sequence identity with mouse miRs was used (miRIDIAN microRNA Hairpin Negative Control #1 IN-001005-01-05). After transfection, cells were harvested, transfection efficiency was tested via flow cytometry, total RNA was extracted and used for subsequent expression analysis by qPCR. Efficient miR downregulation was assessed by measuring expression levels of miR34a-5p and direct miR targets in antimiR transfected cells.

### p53 knockdown

For p53 knockdown in primary lung ECs, a pool of 4 siRNAs targeting the p53 mRNA (ON-TARGETplus Trp53 (22059) siRNA-SMARTpool L-040642-00-0005) was introduced into cells using a lipofectamine based-transfection reagent (LipoFectMax transfection reagent ABP Biosciences FP310). As a negative control, a pool of 4 scrambled siRNA (siGENOME Non-Targeting siRNA Pool #2, 20nmol D-001206-14-20) was tested side by side with the p53 siRNA pool. Briefly, lung ECs of passage 2 were seeded on a 48-well cell culture plate at a density of 5×10^4^ cells/cm^2^ and cultured for 24 h in antibiotic free medium. Thereafter, cells were co-transfected with 100 nM of the pool of 4 siRNAs targeting the p53 mRNA and 50 nM fluorescently labelled control antimiR (miRIDIAN microRNA Hairpin Inhibitor Transfection Control with Dy547 CP-004500-01-05) for 48 h. RNA was isolated 48 h after transfection and analysed via qPCR.

### Total RNA isolation and gene expression analysis

Total RNA including miRs from tissues and cells was isolated using the miRNeasy Mini Kit (Qiagen 217084) and subsequently quantified using NanoDrop Technologies, ND-1000 spectrophotometer. Typically, RNA was isolated from 5×10^4^ -1×10^6^ cells. For gene expression analysis, cDNA was generated using RevertAid reverse transcriptase (Thermo Fisher Scientific). qPCR was performed using the primers listed in Table 1. All reactions were done in technical triplicates using Eppendorf RealPlex 2 Mastercycler with KAPA SYBR Green PCR master mix (Peqlab) and according to the manufacturer’s instructions. Results were normalized to the expression of hypoxanthine-guanine phosphoribosyl-transferase (*Hprt)* and presented as a fold increase relative to *WT* littermate animals based on the ΔΔCt method. To detect intracellular miRs, miScript II RT Kit (Qiagen) and miScript Primer Assays (Qiagen) were used. Intracellular miRs were normalized to small nuclear U6 RNA (snoU6).

**Table 1:** List of primers used for real time PCR analyses.

**Primer pair**	**Sequence**
Hprt Forward	GCAGTCCCAGCGTCGTGATTA
Hprt Reverse	TGATGGCCTCCCATCTCCTTCA
Cccl20 Forward	TCCTTGCTTTGGCATGGGTA
Cccl20 Reverse	TCTTAGGCTGAGGAGGTTCACA
Ctgf Forward	CCTAGCTGCCTACCGACT
Ctgf Reverse	CTTGACAGGCTTGGCGATTT
IL-1 Forward	AGCTCGTCAGGCAGAAGTTT
IL-1 Reverse	TTCTGGCAACTCCTTCAGCAA
Edn1 Forward	ATCTGGGTCAACACTCCCGA
Edn1 Reverse	ACTTTGGGCCCTGAGTTCTT
Trp53 Forward	TCGAGCTCCCTCTGAGCC
Trp53 Reverse	TGGCAGGATATCTTCTGGAGG
p16 Forward	TCGTACCCCGATTCAGGTGATG
p16 Reverse	GCCGGATTTAGCTCTGCTCT
p21 Forward	TGCCAGCAGAATAAAAGGTG
p21 Reverse	TTGCTCCTGTGCGGAAC
Acta2 Forward	GTACCACCATGTACCCAGGC
Acta2 Reverse	GAAGGTAGACAGCGAAGCCA
Wnt7a Forward	CGCTGGGAGAGCGTACTG
Wnt7a Reverse	ATCGCATAGGTGAAGGCAGC
Sirt1 Forward	GATGACAGAACGTCACACGC
Sirt1 Reverse	ACAATCTGCCACAGCGTCAT
Forward HGPS (human specific)	ACTGCAGCAGCTCGGGG
Reverse 1 HGPS (human specific)	AGTTCTGGGGGCTCTGGGT
Reverse 2 HGPS (human specific)	TCTGGGGGCTCTGGGC

### Analysis of EC- and non-EC populations from lung tissues

Lung tissues were dissected from mice >25 weeks, minced and treated with 200 U/ml collagenase type I (Gibco 17100-017) for 45 minutes at 37° C. Thereafter, tissues were triturated using a 19-gauge syringe and tissue homogenate was passed through a 70-μm cell strainer. After centrifugation, the cell pellet was resuspended in cold medium, 10 μl of rat anti-ICAM2 coupled magnetic beads were added and incubated for 30 min. Magnetic separation was used to isolate beads-coupled EC and free non-EC populations with subsequent RNA extraction, gene and miR expression analysis as described above.

### Analysis of heart and lung preparations

For gene expression analysis in tissues, heart and lung extracts were prepared according to [24] and analysed by qPCR. For aortic preparations, aorta was dissected according to previous methods and the whole upper region containing aortic arch was separated from the lower parts of descending aorta [40]. Briefly, all tissues were dissected from >25 weeks old WT and Prog-Tg mice and immediately treated with an RNA stabilization reagent (RNAprotect Tissue Reagent 76106 Qiagen). Thereafter, tissues were transferred to Trizol and lysed using 2.8mm Precellys Zirconium oxide Ceramic beads (Kit CK28) and a Precellys 24 tissue homogenizer according to the manufacturer’s protocols (Bertin Instruments). Lysed tissues were used for total RNA extraction as described above.

### Generation of mRNA and miR libraries and NGS sequencing

RNA samples for intracellular mRNA-and miR sequencing were obtained from lung ECs isolated from ~10-day old mice. Briefly, lung cells were isolated and sorted twice using ICAM-2 magnetic beads to obtain pure ECs. At passage 3, when cells reached 90% confluence, total RNA and miR extracts were prepared and subsequently used for library generation and NGS sequencing. 50 μl EDTA plasma was isolated from three Prog-Tg and WT littermate pairs of >25 weeks old mice. RNA isolation of plasma miRs was performed by TAmiRNA GmbH, Vienna with subsequent miR library preparation using CleanTag™ Small RNA Library Preparation Kit Catalog # L-3206. mRNA and intracellular miR library preparation and sequencing were performed at Vienna Biocenter NGS Facilities. For mRNA analysis, a polyA enrichment was performed from 100 ng total RNA using the NEBNext Poly(A) mRNA Magnetic Isolation Module according to the manufacturer's instructions. Library preparation of polyA+ enriched RNA was performed with the NEBNext UltraII Directional RNA library Kit from Illumina. Quality control of the libraries included a fragment analyser run and a qPCR to determine average size and concentration. The final equimolar pool was sequenced on the Illumina HiSeq2000 with a density of 18 pM and 1% PhiX. miR libraries were prepared from 100 ng total RNA with the QIAseq miRNA library Kit according to the manufacturer's instructions. Libraries were run on a fragment analyser and pooled equimolar by taking the smear concentration from 160-185 bp. The remaining adapter dimers were cleared from the pool by gel extraction on the Pippin Prep (3% agarose) with a tight cut from 155 bp to 190 bp. The concentration of the final pool was determined by qPCR using the KAPA library quant Kit and sequencing was performed on the Illumina HiSeq2000 with a density of 18 pM and 1 % PhiX. Bioinformatic analysis for all libraries was performed by the VBCF bioinformatics and scientific computing facility.

### Bioinformatics analyses

The short sequencing reads were aligned against the *mus musculus* reference genome (GRCm38 release) [58] with STAR [59], version 2.5.1b using 2-pass alignment mode. For mRNA and intracellular miR libraries, roughly 30 million short reads per replicate were generated, of which ~80% and ~55-75%, respectively, could be mapped to the reference genome. For plasma miR libraries ~15-30 million short reads were generated with a mapping score of 15% and 5%, respectively. Gencode v4 annotation was used in the alignment. After alignment, the short reads were associated with known genes, and the number of reads aligned within each gene was counted using HTSeq tool [60] version 0.5.4p3. The data was normalised to remove variation between samples caused by non-biological reasons and to make the values comparable across the sample set using the TMM normalisation method of the edgeR [61], R/Bioconductor package (R version 3.3.2, Bioconductor version 2.12). The method takes the variable number of total reads across samples into account by calculating specific scaling factors between the samples. For differential expression analyses, the data were further log transformed using the voom [62] approach, and the R package limma [63] was used to perform the statistical testing. Significance thresholds applied were a p-value<0.05 and a minimum expression fold change of 1.5 between the compared sample sets for mRNA data and a minimum fold change of 1 for miR data. For detection of functional enrichment in the differentially expressed gene lists (DE list enrichment) and for detection of functional enrichment towards the top of the list when all genes have been ranked according to the evidence for being differentially expressed (Ranked list enrichment), the GO [64, 65], KEGG [66] and Reactome [67] databases were used, together with topGO (Alexa A, Rahnenfuhrer J (2021). topGO: Enrichment Analysis for Gene Ontology. R package version 2.46.0.), Gostats [68], and GAGE [69] packages in R/Bioconductor. Heatmaps in Supplementary Figure 2C were generated with R. To generate the bar graph in Supplementary Figure 3A the top 200 GO terms from GAGE analysis were used for both datasets. The KEGG (Kyoto Encyclopedia of Genes and Genomes) pathway enrichment analysis of the differentially expressed miRs was conducted by the miRSystem (http://mirsystem.cgm.ntu.edu.tw/index.php) online database tool. To identify potentially enriched pathways, 41 significantly upregulated miRs and their fold changes were submitted to the miRSystem database and the pathway list was analysed by adjusting parameter settings of 50–500 genes in biological functions/pathways, Hit ≥ 5.0, O:E ratios ≥ 2.0.

### Immunofluorescence in tissues

For immunofluorescence analysis, lungs and hearts of >25 weeks aged mice were fixed overnight in 4% formaldehyde in PBS, dehydrated in xylene and embedded in paraffin. Serial 3-μm sections were deparaffinised, rehydrated followed by antigen retrieval using pressure cooker method performed for 30 min in Tris 0.01M, EDTA 0.001M. Then samples were quenched in 0.1% glycine in PBS, permeabilized and blocked in PBS with 2% BSA, 0.1% Tween for 1 h at RT and stained with the following primary antibodies: rabbit anti-CDKN2A/p19ARF 1:100 (ab80, Abcam), rabbit anti-PECAM-1/CD31 1:30 (LB-B4737, LifeSpan BioScience) and rat anti-CD3 1:400 (MCA1477, Bio-Rad). Next samples were incubated with secondary antibodies: goat anti-rabbit 488 (ThermoFisher), goat anti-rabbit 594 (ThermoFisher) and donkey anti-rat (SA5-10028, ThermoFisher) all diluted 1:200 and DAPI nucleic acid stain (1:500 in PBS) and mounted in Mowiol 4-88 (Sigma-Aldrich).

### BrdU incorporation

Bromodeoxyuridine (BrdU) incorporation assay was performed using the APC BrdU Flow Kit (557892BD, Pharmingen). ECs at passage 2 were seeded in triplicates on 48-well plates at a density of 4 × 10^4^ cells/cm^2^ and cultured for 24 h prior to BrdU addition. Incorporation of BrdU was performed over 40 h and thereafter BrdU positive cells were measured by flow cytometry. The percentage of BrdU positive cells is calculated according to the total cell number measured by flow cytometry.

### Plasma collection, conditioned media and enzyme-linked immunosorbent assay (ELISA)

Lung ECs at passage 2 were seeded at a density of 5 x 10^4^ cells/cm^2^ on a 24-well cell culture plate in phenol red-free complete culture medium (21063029, Gibco). After 48 h of incubation, the conditioned medium was collected, centrifuged at 5000 x g and filtered through a 0.2 μm filter. For negative control, the corresponding medium was incubated for the same period in the absence of cells. For plasma collection, blood from mice at the age >25 weeks were collected and spun down at 1900 x g followed by centrifugation at 16000 x g at 4° C for 10 min. Thereafter plasma was shock frozen in EDTA-overlaid tubes and stored at -80° C until used. ELISA assay was performed using Quantine ELISA Mouse CCL20/MIP-3α Immunoassay kit (MCC200, R&D Systems) and according to manufacturer’s instructions. At least three biological replicates were used and each tested in technical duplicates. Two positive controls for ELISA were included: i) ECs treated with 20 μg/ml TNFα (PMC3014, ThermoFisher) and ii) ELISA kit provided control (892547, mouse Mip-3 α). Optical density was measured on a microplate reader at 450 nm and at 540 nm for wavelength correction.

### SDS-PAGE, immunoblotting and antibodies

For direct lysis, cells were washed twice with PBS, and thereafter covered with 50 mM Tris-HCl, 100 mM DTT, 2% SDS, 0.1% bromophenol blue and 10% glycerol pH 6.8. Cell lysates were separated by SDS-PAGE, transferred onto nitrocellulose membranes, followed by a blocking step and incubation with primary antibodies as described previously [24]. To visualize protein bands, membranes were incubated with peroxidase-coupled secondary antibodies (Jackson Laboratories) for 1 h with subsequent detection using the SuperSignal West Pico Plus Chemiluminescent Substrate (34580, Thermo Scientific). Protein bands were quantified using ChemiDoc MP Imaging System using Image Lab Software. Following primary antibodies were used: rabbit anti-PECAM1 (LS-B4737, LSBio), mouse monoclonal antibody (mAb) against human lamin A+C (Chemicon, clone JoL2, mab3211, Abcam) for detection of human lamins (including human progerin), mouse monoclonal Lamin A/C antibody E-1 (Santa Cruz sc-376248) for detection of mouse and human lamins, and mouse mAb against α-tubulin (clone B-5-1-2, T5168, MilliporeSigma).

### Statistics

Data are presented as the mean ± SEM or median with minimum and maximum values. All experiments using WT and Prog-Tg ECs were performed at least 3 times using different primary cells isolated from corresponding littermate pairs. Statistical analyses were performed on dCT values using GraphPad Prism statistical software. The two groups were analysed using the unpaired two-sided Student’s t-test and paired *t* test was used in experiments with littermate pairs. For evaluations of non-normally distributed data, a nonparametric Mann-Whitney rank-sum test was used. One-way ANOVA was used for multiple comparisons (Kruskal-Wallis test). Data were considered statistically significantly different if *p*< 0.05.

### Study approval

Animal studies were approved by the Regional Ethics Committee for Laboratory Animal Experiments at the Medical University of Vienna and the Austrian Ministry of Science Research and Economy (BMWFW-66.009/0321-WF/V/3b/2016 and BMWFW-66.009/0156-WF/V/3b/2017).

## Supplementary Material

Supplementary Figures

Supplementary Table 1

Supplementary Table 2
