# Supplementary material for: Endothelial and systemic upregulation of miR-34a-5p fine-tunes senescence in progeria
Source: Aging (Albany NY). 2022 Jan 12;14(1):195–224. doi: 10.18632/aging.203820 (PMC8791216; doi:10.18632/aging.203820)
Supplement: Supplementary Figures [file aging-14-203820-s001.pdf]

SUPPLEMENTARY FIGURES

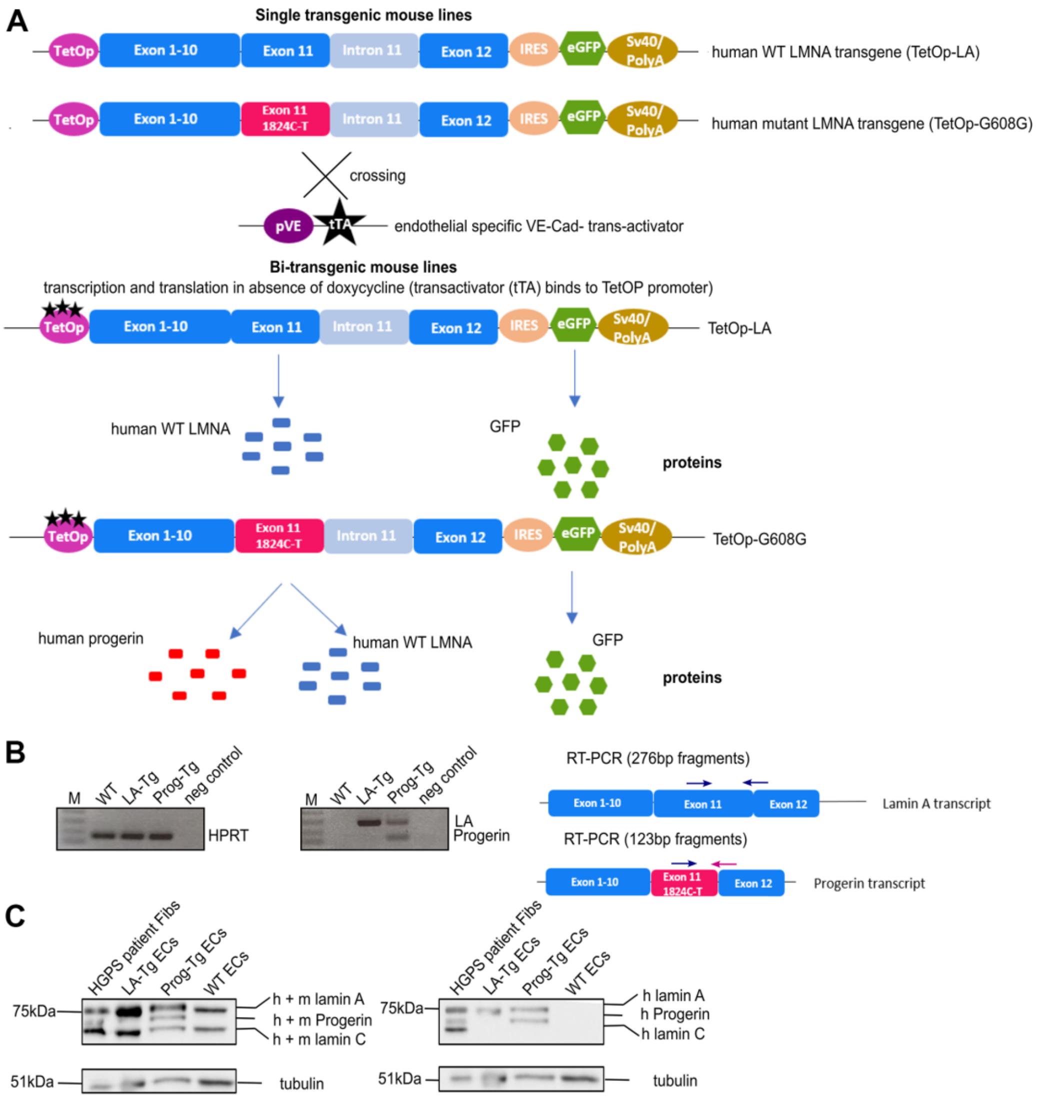

Supplementary Figure 1. Organization of the human wildtype and mutant lamin A minigenes and generated transcripts and proteins. (**A**) Schematic representation of the human wildtype (TetOp-LA) and mutant (TetOp-G608G) lamin A minigene carrying the most common HGPS mutation (1824C>T). Transgenic mice harboring these transgenes were crossed with mice expressing the transactivator under the endothelium-specific VE-Cad promoter (pVE-tTA) to generate bi-transgenic mice expressing the human wildtype or mutant (progerin) lamin A in the endothelium. Proteins encoded by these minigenes are indicated below. Human lamin A minigenes harbor complete exon 1-10, exon 11, with or without HGPS mutation (1824C>T), intron 11, exon 12, internal ribosomal entry site (IRES), coding region for eGFP and SV40 poly A site as indicated. TetOp indicates the tetracycline-responsive operator. pVE indicates the endothelial specific VE-cadherin (*Cdh5*) promoter and tTA (black stars) indicate the tetracycline trans-activator. (**B**) RT-PCR analyses of transcripts in WT, LA-Tg and Prog-Tg lung endothelial cells, using primers from [33] that specifically amplify human *LMNA* sequences (right panel), show one band of expected 276 bp size for the WT human *LMNA* (LA) and one band of expected 123 bp size for human progerin and no bands in control WT samples and negative controls. RT-PCR analysis using mouse *Hprt* specific primers shows expected band size of 172 bp. Right panel shows a schematic representation of the triple RT-PCR and primer binding sites in *LMNA* minigene. (**C**) Immunoblot analyses of cell extracts of mouse WT, LA-Tg and Prog-Tg endothelial cells and of human HGPS patient fibroblasts as control, using lamin A/C antibody E-1, (Santa Cruz sc-376248) detecting endogenous mouse lamin A/C and ectopic human lamin A and progerin (left panel) and monoclonal antibody clone JoL2 (Chemicon, mab3211, Abcam) detecting exclusively human lamin A/C and progerin. Tubulin was used as loading control. Note that the TetOp-G608G transgene generates both wildtype and mutant lamin A transcripts and proteins, while the TetOp-LA transgene produces exclusively wildtype lamin A.

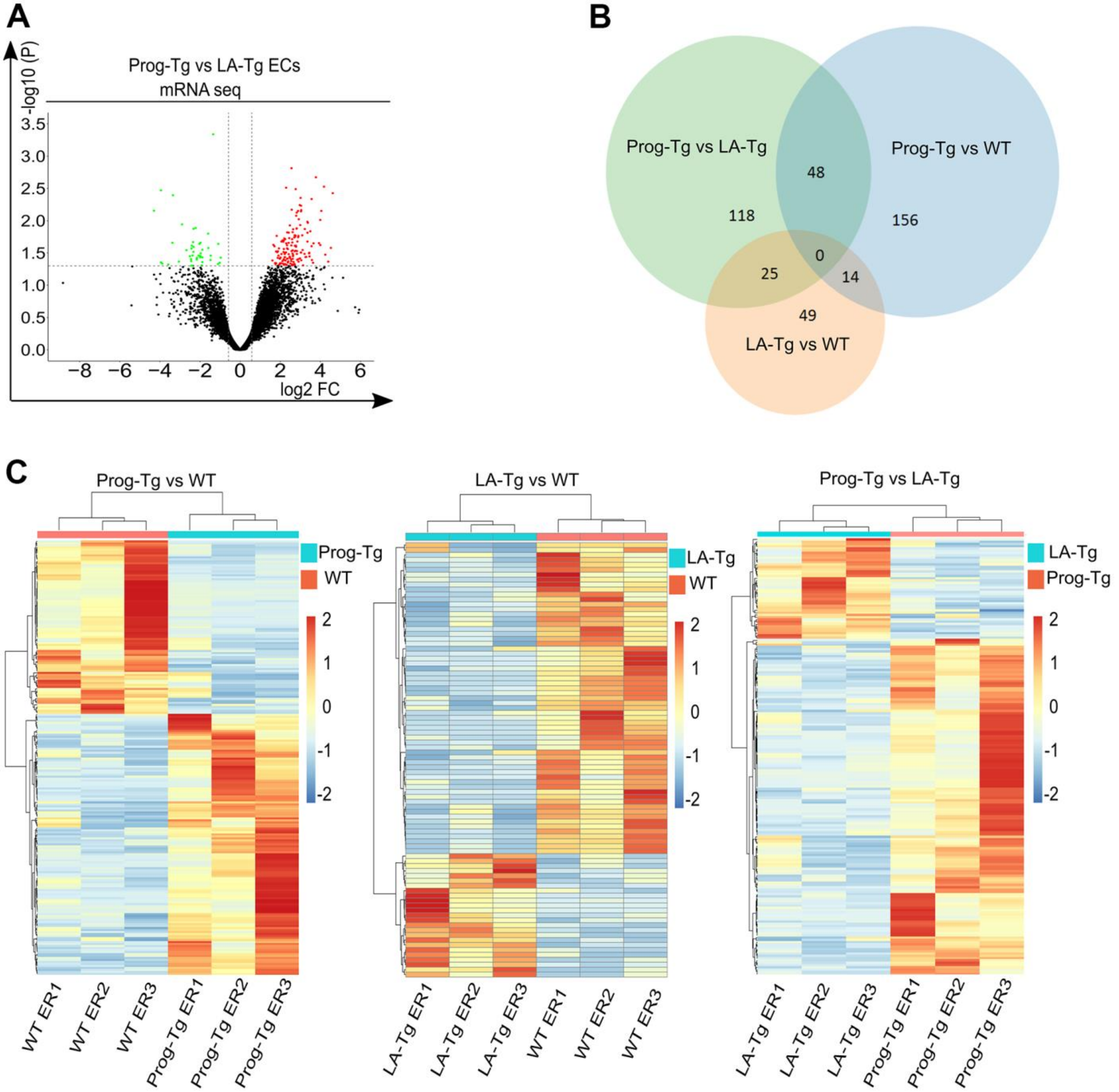

Supplementary Figure 2. Differential gene expression analyses of Prog-Tg and LA-Tg endothelial cells. (**A**) Volcano plot depicting differential expression (DE) analysis of genes in Prog-Tg/LA-Tg lung endothelial cells. Red, upregulated, green, downregulated genes; X-axis, $\log_2$ values of fold change (FC>1,5 and <-1.5) and y-axis, $-\log_{10}$ values of p-value ($p<0.05$, n=3). (**B**) Analysis of shared DE genes between Prog-Tg/WT, LA-Tg/WT and Prog-Tg/LA-Tg depicted by a 3-way Venn diagram. (**C**) Heatmaps showing clustering of the differentially expressed genes in Prog-Tg vs WT and LA-Tg vs WT (associated with Figure 1A) and of Prog-Tg vs LA-Tg (associated with Supplementary Figure 2B).

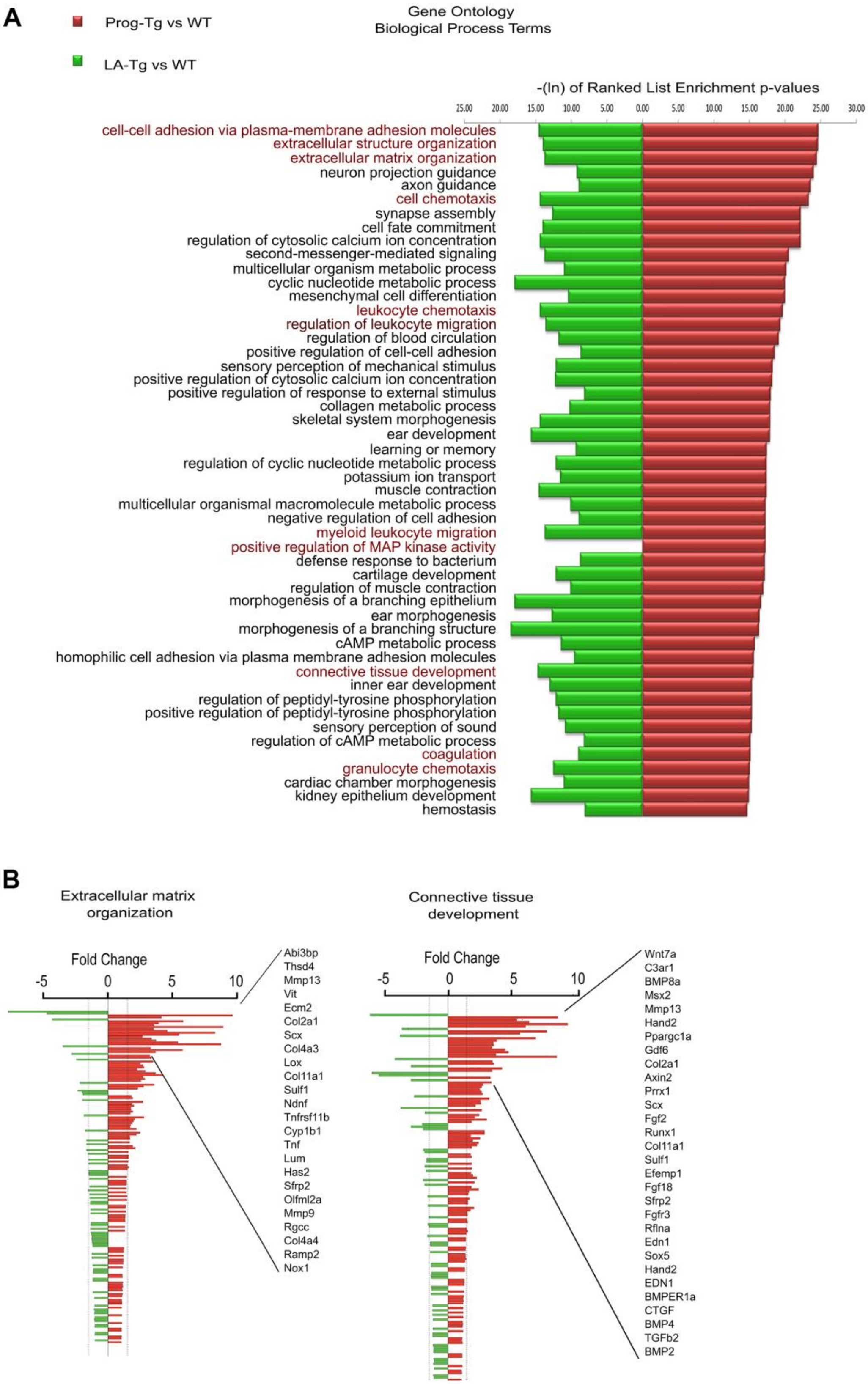

Supplementary Figure 3. Gene ontology analyses of differentially expressed genes. (**A**) Depiction of the top 50 gene ontology biological process terms as found in the ranked list enrichment analysis of Prog-Tg/WT compared to LA-Tg/WT DE genes. The x-axis represents -ln of ranked list enrichment p-values. (**B**) Ranked-list enrichment gene ontology analysis in Prog-Tg/WT lung ECs showing deregulated genes in terms “extracellular matrix organization” and “connective tissue development”.

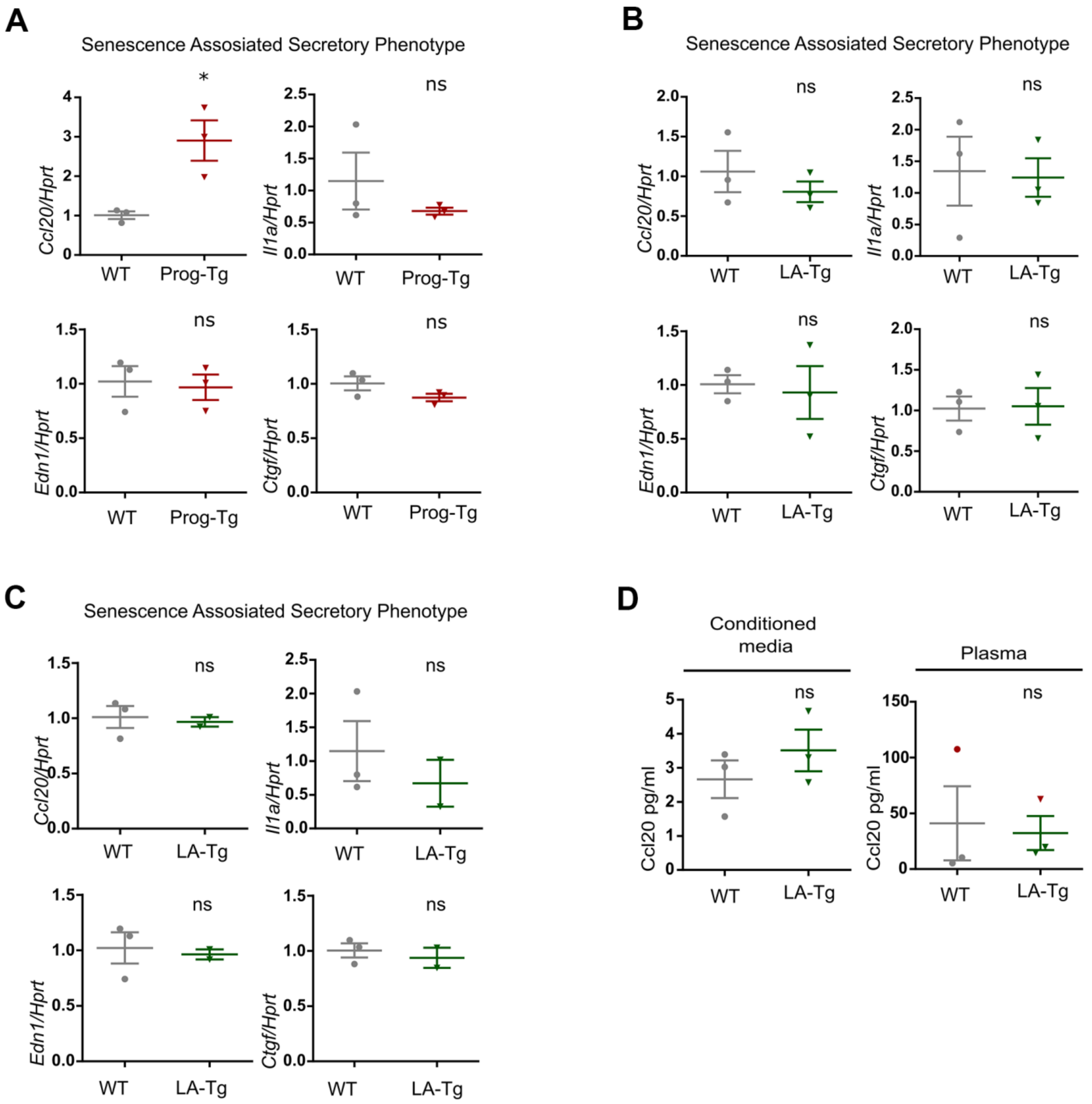

Supplementary Figure 4. Expression analyses of senescence and SASP markers in endothelial cells, conditioned media and plasma. (**A**) Gene expression analysis of pro-inflammatory markers *Ccl20* and *Il-1a* and pro-fibrotic markers *Edn1* and *Ctgf* in WT and Prog-Tg ECs derived from heart tissue. (**B**, **C**) Gene expression analysis in WT and LA-Tg lung derived ECs (**B**) and heart derived ECs (**C**). (**D**) ELISA assay for detection of Ccl20 in cell culture supernatant samples of lung ECs and in plasma samples of >25 week-old WT and LA-Tg mice, respectively. Red sphere and rectangle in the left panel represent two littermate mice with unusually high levels of Ccl20. Paired two-tailed Students *t* test was applied for *in vivo* experiments with littermate pairs and unpaired two-tailed Students *t* test for *in vitro* co-culture experiments. ns=non-significant, $*p<0.05$.

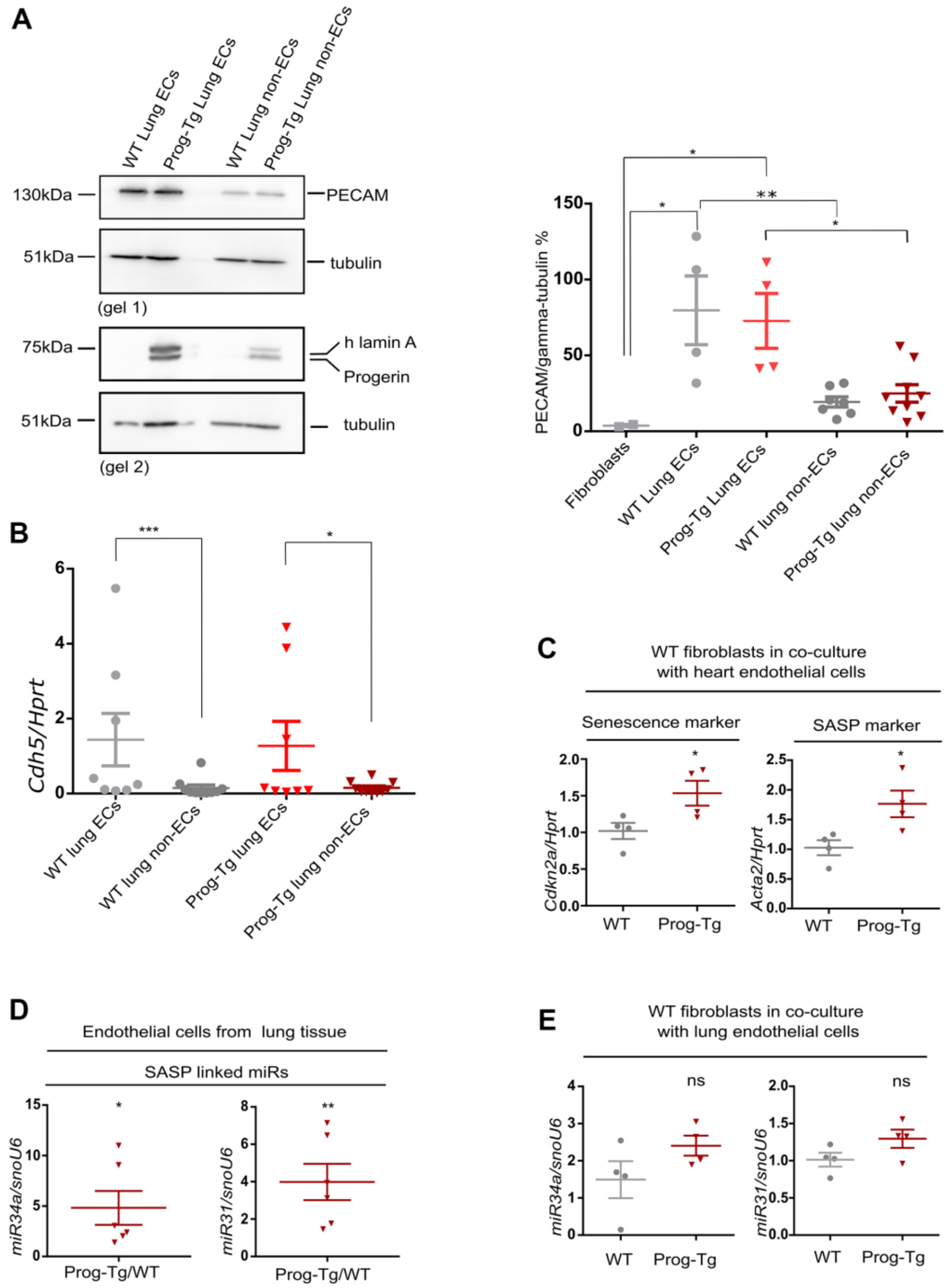

Supplementary Figure 5. Expression analyses of SASP and senescence markers in endothelial and non-endothelial cells from tissue and in co-cultures of fibroblasts and endothelial cells. (**A**) Endothelial cell- and non-endothelial cell populations obtained from lung homogenates using ICAM-2 magnetic bead separation were tested for purity using immunoblotting with endothelial cell marker PECAM-1 (gel 1), anti-human lamin A to detect progerin (gel 2) and respective gamma-tubulin loading controls. Quantification of PECAM-1/gamma-tubulin protein band intensities in EC and non-EC populations (right panel). Immortalized fibroblasts (not shown on representative blot) were used as a negative control. Note that the low PECAM-1 levels in non-EC populations indicate the removal of the majority of ECs. For EC and non-EC populations n=4-9. PECAM-1-negative fibroblasts were used as negative control (n=2). For statistical evaluation, one-way ANOVA was used (Sidak's multiple comparisons test). (**B**) EC and non-EC populations in (**A**) were tested for gene expression levels of EC marker VE-cadherin (*Cdh5*) by qPCR. (**C**) WT fibroblasts were co-cultured with ECs derived from hearts of WT and Prog-Tg mice, respectively, and tested for gene expression of senescence marker *Cdkn2a* and pro-fibrotic marker *Acta2* by qPCR. (**D**) Expression of SA-miRs, miR34a-5p and miR31-5p were tested in EC populations of Prog-Tg versus WT mice (>25 weeks, n≥3). (**E**) Expression of miR-34a-5p (miR-34a) and miR-31-5p (miR-31) in WT fibroblasts co-cultured with either WT or Prog-Tg lung ECs. For qPCRs n=4-6. Paired two-tailed Students *t* test was used for *in vivo* experiments and an unpaired two tailed Students *t* test for *in vitro* co-culture experiments. ns=non-significant, *p<0.05, **p<0.01, ***p<0.001.

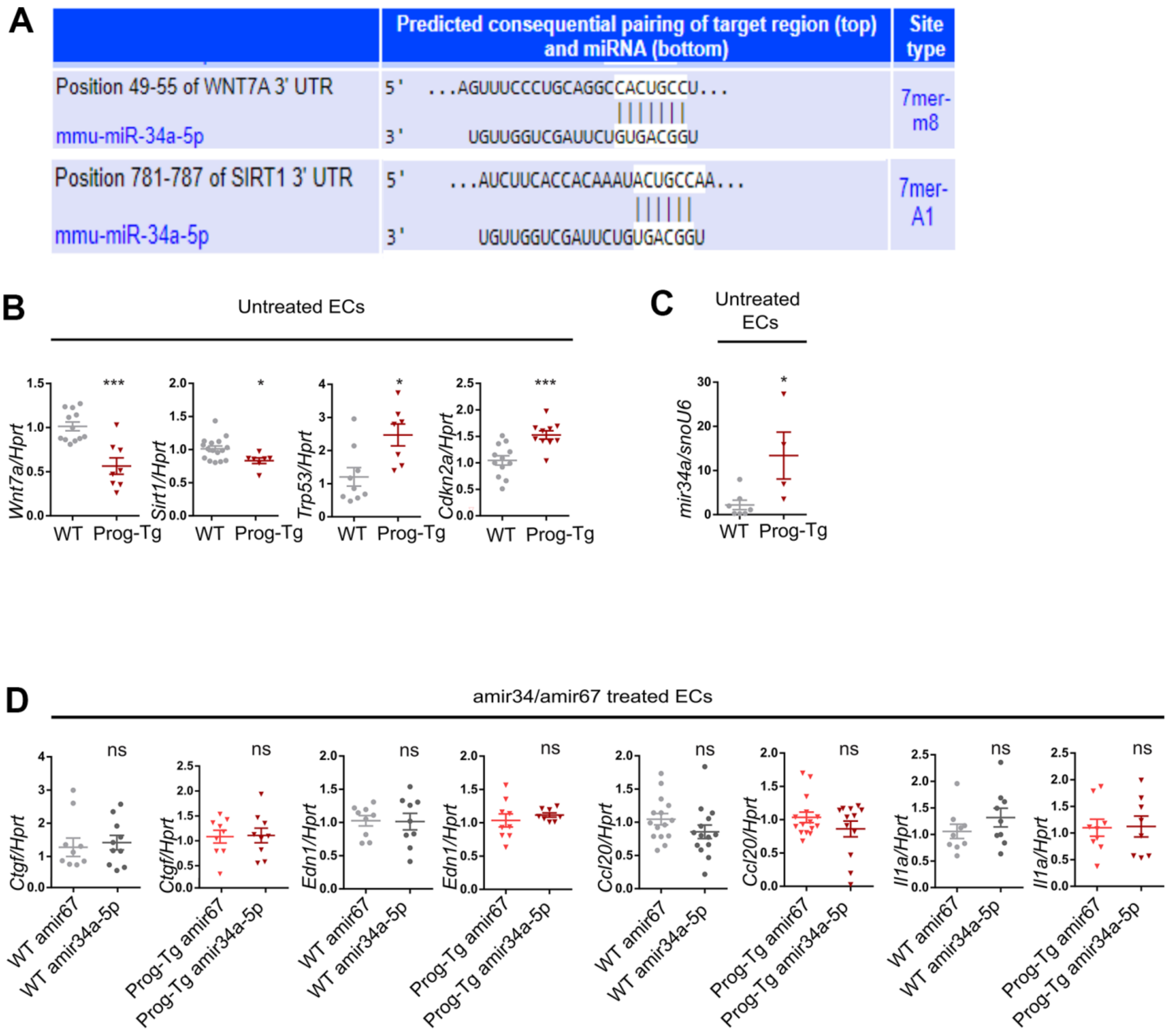

Supplementary Figure 6. Anti-miR34 treatment of endothelial cells. (**A**) Image showing binding sites of miR34a-5p in 3´ UTR of *Wnt7a* and *Sirt1* (TargetScaMouse version 7.2). (**B**) Gene expression analysis of depicted targets in untreated WT and Prog-Tg lung ECs. (**C**) Gene expression analysis of miR34a-5p in untreated lung ECs used for antimiR34 transfection experiments. (**D**) Gene expression analysis of depicted genes in antimiR34a-5p versus control antimiR67 transfected WT and Prog-Tg lung ECs. For qPCR n≥3, statistics were performed using unpaired two-tailed Students *t* test, ns=non-significant, *p<0.05, ***p<0.001.
